# An integrative meningioma surfaceome atlas and a transferable framework for CAR-T target nomination

**DOI:** 10.3389/fbinf.2026.1919802

**Published:** 2026-10-05

**Authors:** Vivek Sanker, Dhanya Mahesh, Linda Liverani, Matthew Alexander Abikenari, Zhikai Li, Yi Zhang, Debebe Theodros, Ravi Medikonda, Maria Jose Cavagnaro, Matei Alexandru Banu, Atman Desai, Michael Lim

**Affiliations:** 1 Department of Neurosurgery, Stanford University, Stanford, CA, United States; 2 Geisel School of Medicine, Dartmouth College, Hanover, NH, United States; 3 Department of Clinical Neurosciences, Addenbrooke’s Hospital, Cambridge, United Kingdom

**Keywords:** bispecific CAR-T, CAR T-cell therapy, membrane topology, meningioma, on-target off-tumor toxicity, single-cell RNA sequencing, surfaceome, tumor-associated antigen

## Abstract

Meningioma is the most common primary intracranial tumor, yet recurrent and high-grade disease has few durable systemic options, and, unlike hematological malignancies, no systematically filtered cell-surface antigen resource exists to guide chimeric antigen receptor (CAR) T development. We address this gap not by nominating a trial-ready target but by building two reusable resources: an integrative meningioma surfaceome atlas and a transferable, gate-based framework that separates transcriptomic discovery from CAR-T target nomination. Analyzing single-cell RNA sequencing from eight meningioma patients (GSE183655; 80,220 quality-controlled cells), patient-aware differential expression followed by a curated surfaceome filter (UniProt plasma-membrane localization; extracellular domain ≥20 aa; exclusion of ribosomal, nuclear, cytoplasmic, and secreted proteins) reduced 83 scored candidates to 13 *bona fide* plasma-membrane (Tier A) antigens, removing prominent false positives such as the nuclear proteins FOXC1 and MGMT, the cytoplasmic enzyme lactate dehydrogenase A (LDHA), and the secreted protein PTGDS. Because expression ranking establishes discovery but not CAR-T suitability, we then applied explicit nomination gates: a systemic-safety score integrating whole-body GTEx transcriptomics with Human Protein Atlas protein-level evidence; a membrane-topology tractability gate distinguishing antibody-accessible single-pass and GPI-anchored ectodomains from multi-pass transporters and tail-anchored proteins; and a safety × coverage metric. Under stringent thresholds (combined safety ≥0.55, tumor-cell detection fraction ≥50%, safety × coverage ≥0.30), no Tier A antigen qualified. Ubiquitously expressed antigens were correctly demoted, including the tetraspanin CD9 (53.2% tumor-cell coverage but pan-tissue protein expression and high plasma abundance), and the highest-ranked transporter SLC7A2 was demoted by multi-pass topology (largest extracellular loop 38 aa) despite favorable transcriptomic safety. Leukemia inhibitory factor receptor (LIFR), a single-pass type I receptor with a contiguous 789-aa ectodomain, was the only antibody-tractable nominee for experimental follow-up yet remained liability laden (combined safety 0.41; safety × coverage 0.135) and is not a validated target. Cross-dataset expression overlap (93.3%) reflected mRNA reproducibility, not CAR-T validity or clinical validation, and generic-antibody docking was exploratory only. All findings are transcriptomic and require surface-level confirmation. This work contributes a reproducible meningioma surfaceome atlas and a transferable nomination framework and shows that rigorous gating leaves no single antigen with a favorable safety × coverage × tractability profile, motivating bispecific and logic-gated CAR-T strategies.

## 1 Introduction

Meningiomas are the most common primary intracranial tumors in adults, accounting for roughly a third of all central nervous system (CNS) neoplasms (Ostrom et al., 2023). Most are benign and surgically curable, but recurrent, atypical (WHO grade 2), and anaplastic (grade 3) tumors frequently progress despite resection and radiotherapy, and effective systemic therapies are lacking (Goldbrunner et al., 2021; Louis et al., 2021). This unmet need has prompted interest in immunotherapeutic strategies, including chimeric antigen receptor (CAR) T cells.

CAR-T therapy has transformed the treatment of B-cell malignancies, where lineage-restricted antigens such as CD19 provide homogeneous, surface-accessible targets (June and Sadelain, 2018). Translating this success to CNS solid tumors has proven far harder: early trials against EGFRvIII, IL13Rα2, and GD2 in glioma have shown that antigen heterogeneity, antigen-negative escape, restricted trafficking, and on-target, off-tumor toxicity are formidable barriers (O'Rourke et al., 2017; Brown et al., 2016; Majzner et al., 2022). The single most important determinant of a feasible solid-tumor CAR-T program is the target antigen itself, which must be tumor-enriched, expressed on a high fraction of malignant cells, displayed at the cell surface with an antibody-accessible epitope, and, critically, largely absent from vital normal tissues (MacKay et al., 2020).

Each requirement is a distinct failure mode. Heterogeneous expression permits antigen-negative escape and relapse (Majzner et al., 2018). Shared expression with normal tissue can cause severe or fatal on-target off-tumor toxicity, as seen when CAR-T cells targeting ERBB2/HER2 or carbonic anhydrase IX encountered antigen on healthy epithelium (Morgan et al., 2010; Lamers et al., 2013). Because CAR-T cells circulate systemically, normal-tissue assessment must span the whole body and include protein-level evidence, not only transcript abundance in a single organ. Not every membrane protein is a viable antibody target: single-pass type I/II receptors and GPI-anchored proteins expose contiguous ectodomains suitable for antibody and CAR binding, while multi-pass transporters and tail-anchored proteins present only short extracellular loops that are poor antibody targets (MacKay et al., 2020).

Single-cell transcriptomics offers an attractive route to nominate candidate antigens, but *in silico* discovery is only as reliable as its filters. Transcript abundance does not establish surface-protein display; transmembrane-helix prediction alone admits intracellular, nuclear, and secreted proteins; brain-restricted safety screening ignores systemic exposure; and docking against a non-cognate antibody cannot establish target suitability. Curated resources now make rigorous filtering feasible, including mass-spectrometry and *in silico* surfaceome catalogs (Bausch-Fluck et al., 2015; Bausch-Fluck et al., 2018), protein-level tissue atlases (Uhlén et al., 2015), and whole-body normal-tissue transcriptomes (GTEx Consortium, 2020). Meningioma has been characterized at single-cell resolution and by genomic profiling (Clark et al., 2013), but a systematically filtered, CAR-T-oriented surface-antigen resource is lacking.

Here, we present an integrative single-cell pipeline that converts meningioma transcriptomic discovery into a quality-gated surfaceome atlas. Beyond expression ranking, we impose explicit nomination gates, curated surfaceome localization, whole-body GTEx, and Human Protein Atlas systemic safety, membrane-topology tractability, and a safety × coverage metric, and report a relative gated-rank score that is explicitly not an absolute feasibility probability. We frame all outputs as hypothesis-generating, and we show that rigorous gating both removes confident false positives and reveals that no single antigen in this cohort is clearly favorable, thereby motivating multi-antigen strategies such as bispecific or logic-gated CAR-T designs (Hegde et al., 2016; Roybal et al., 2016; Cho et al., 2018).

## 2 Methods

All analyses were performed using custom Python pipelines (Figure 1) available on GitHub (See Data Availability Statement). The methods below describe each computational stage with thresholds, equations, and software versions. Extended derivations and additional hyperparameter grids are provided in Supplementary Material.

**FIGURE 1:**
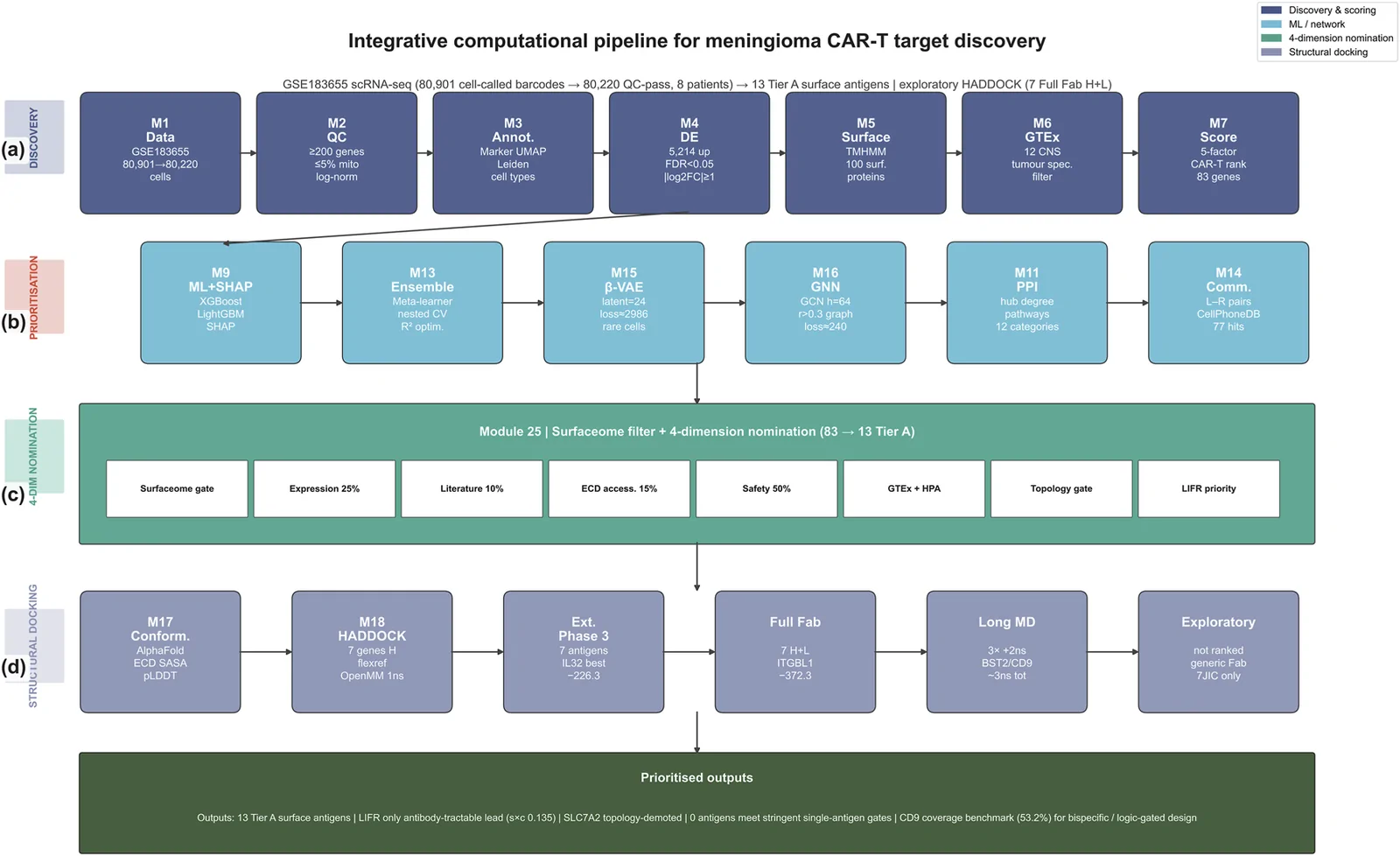
Integrative computational pipeline for meningioma CAR-T target discovery (Modules 1–25). **(a)** Discovery layer (Modules 1–7): GSE183655 scRNA-seq ingress (80,901 Cell Ranger barcodes → 80,220 QC-pass cells; 99.2% retention; six patients, eight meningioma libraries); QC and log-normalization (≥200 genes/cell; ≤5% mitochondrial reads); marker-based UMAP/Leiden annotation; patient-stratified differential expression (5,214 consistently upregulated genes, Benjamini–Hochberg FDR <0.05, |log_2_FC| ≥ 1 in ≥2/6 patients); TMHMM surface-protein filter (100 candidates); GTEx CNS tumor-specificity filter; five-factor discovery score (expression 20%, specificity 30%, tumor-cell detection fraction 20%, immunogenicity 20%, escape 10%) yielding 83 ranked genes. **(b)** Prioritization layer (Modules 9–16): LightGBM/XGBoost ensemble with SHAP attribution; stacked meta-learner with nested cross-validation; β-VAE rare-cell module (latent dimension 24; final loss ≈2,986); convolutional network on co-expression graph (80 genes, Pearson r > 0.3; final loss ≈240.4); PPI/pathway hub annotation; Cell PhoneDB ligand–receptor screening (77 pairs). These ML/network outputs provide exploratory context and are not used directly for final CAR-T nomination. **(c)** Four-dimensional nomination layer (Module 25): Curated surfaceome filter reducing 83 input genes to 13 Tier A *bona fide* plasma-membrane antigens (70 excluded); gated score combining safety 50% (GTEx v8 whole-body + HPA v25.1 protein), expression 25%, literature 10%, and extracellular-domain accessibility 15%; topology and ubiquitous-protein filters applied. **(d)** Structural docking layer (Modules 17–24; exploratory, excluded from ranking): AlphaFold ECD accessibility; HADDOCK3 rigid-body/flexref with a generic anti-CD19 Fab surrogate (PDB 7JIC); full Fab H + L models for a seven-antigen cohort; and short OpenMM molecular-dynamics extensions. Prioritized outputs: 13 Tier A surface antigens; LIFR sole antibody-tractable lead (safety × coverage 0.135; gated relative rank 0.619); SLC7A2 topology-demoted despite highest transporter safety × coverage (0.245); zero genes meet stringent single-antigen gates (safety ≥0.55 and detection fraction ≥50%); CD9 (53.2% detection fraction) retained as bispecific coverage benchmark.

### 2.1 Study design and datasets

This retrospective computational study analyzed publicly deposited transcriptomic data without collecting new human samples. The discovery cohort GSE183655 (single-cell RNA-seq, eight meningioma patients; GEO) was complemented by three independent expression-reproducibility cohorts (GSE136661, bulk RNA-seq; GSE16581 and GSE165341, microarray) to assess reproducibility of the mRNA-based ranking, not to provide clinical or CAR-T validation. Ethical approval for the original data generation resides with the depositing investigators; our analyses used only de-identified public matrices.

Raw Cell Ranger count matrices for GSE183655 were retrieved from NCBI GEO (gene-level Ensembl identifiers in features. tsv; 80,901 cell-called barcodes) and were used for the primary discovery atlas (quality control (QC), annotation, differential expression, and nomination gates). Separately, to eliminate annotation mismatch with GTEx v8, raw SRA FASTQs for all 15 runs of SRP336139/PRJNA761582 were re-quantified against GENCODE v26 with kallisto|bustools (10x Chromium v3); barcodes were transferred from the deposited annotated atlas for tumor-versus-microenvironment reporting on this common reference (Supplementary Figure S10; Supplementary Tables S4a and S4b). After quality control of the deposited matrices, 80,220 cells were retained (99.2% pass rate); the published author-reported count (57,114 cells) reflects a different filtering scheme. The code is available on GitHub (See Data Availability Statement). Random seeds were fixed (random_state = 42) for machine-learning splits.

### 2.2 Single-cell RNA-seq processing and cell-type annotation

Quality control was performed in Scanpy v1.9 (Modules 1–2). Cells were retained if they expressed ≥200 genes, had ≤5% mitochondrial read fraction and ≤50% ribosomal read fraction; genes detected in <3 cells were removed. After filtering, 80,220 cells and 26,500 genes were retained (Supplementary Figure S1; median 2,913 genes/cell). Counts were normalized to 10,000 counts per cell and log-transformed. Highly variable genes (n = 2,000; flavor = seurat_v3) informed PCA (50 components) and a neighbor graph (n_neighbors = 15). Leiden clustering (resolutions 0.5, 1.0, 1.5) and curated marker panels resolved meningioma, immune, stromal, vascular, and pericyte compartments (Figure 2). Meningioma markers use the HGNC symbol MUC1 (clinical alias Epithelial Membrane Antigen (EMA)). Per-candidate expression of leukemia inhibitory factor receptor (LIFR), SLC7A2, VAMP5, SLC16A1, and CD9 by annotated cell type is shown in Supplementary Figure S8 and Supplementary Table S7. Orthogonal Copy number variant (CNV) confirmation (inferCNV/CopyKAT) was not completed in this revision because genomic coordinates are absent from the processed AnnData (Supplementary Material).

**FIGURE 2:**
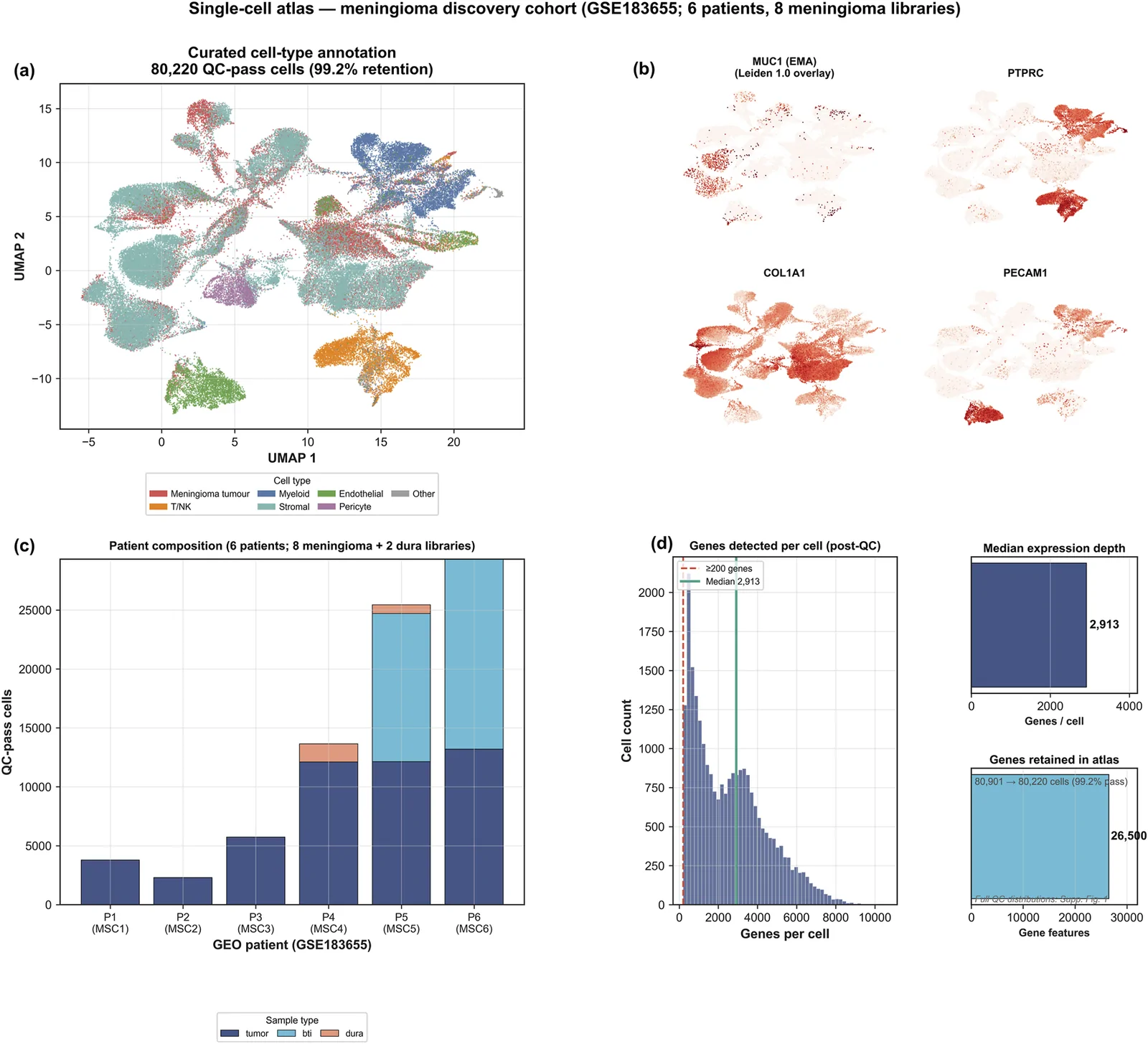
Single-cell atlas of the meningioma discovery cohort (GSE183655; six patients, eight meningioma libraries). **(a)** UMAP of 80,220 QC-pass cells (from 80,901 cell-called barcodes; 99.2% retention) colored by curated, grouped cell-type annotation: meningioma tumor (n = 22,427), stromal (36,006), myeloid (8,578), T/NK (5,485), endothelial (4,977), pericyte (2,074), and other/unknown (673). Cell-type key in dedicated legend strip below panel. Tumor cells form patient-structured clusters supporting patient-stratified differential expression. **(b)** Leiden clustering (resolution 1.0) with marker-gene expression overlay on shared UMAP coordinates for MUC1 (clinical alias EMA), PTPRC, COL1A1, and PECAM1. Expression intensity scaled per gene (light gray = low; red = high). **(c)** Patient composition stacked bar chart for six GEO patients (Patient 1–6; MSC1–MSC6) showing QC-pass cell counts by sample type: primary tumor (dark blue), brain–tumor interface/BTI (light blue), and matched dura (orange). All six patients contribute cells, for a total of ten GEO libraries (eight meningioma (tumor or BTI) plus two dura). **(d)** Atlas QC metrics: left, histogram of genes detected per cell with minimum threshold (red dashed, ≥200 genes/cell) and pipeline median (green line, 2,913 genes/cell); right, horizontal bars for median expression depth (2,913 genes/cell) and genes retained (26,500 features), with barcode retention (80,901 → 80,220; 99.2% pass). Full QC distributions in Supplementary Figure S1. Priority-antigen expression by cell type: Supplementary Figure S8 and Supplementary Table S7.

Patient-stratified differential expression (Module 4) compared annotated meningioma tumor cells versus non-tumor cells from the same cohort (predominantly tumor-microenvironment and dura-associated compartments present in GSE183655), using two-sided moderated t-tests with Benjamini–Hochberg false discovery rate (FDR) correction genome-wide. This is a tumor-versus-microenvironment contrast within the discovery cohort, not a tumor-versus-matched normal meningeal/arachnoidal tissue comparison; systemic normal-tissue safety is assessed separately and allogeneically via GTEx/Human Protein Atlas (HPA). Genes with FDR <0.05 and |log_2_FC| ≥ 1.0 in ≥2 of 8 patients were classified as consistently upregulated (5,214 genes). TMHMM transmembrane prediction and GTEx tumor specificity yielded 100 initial surface-protein candidates (Modules 5–6). GENCODE v26/GTEx v8-matched Ensemble transcript (ENST) identifiers for narrative antigens, corrected where prior pins mapped to the wrong gene, and transcript-level GTEx median TPM are reported in Supplementary Tables S4a and S4b (revised from the official RSEM transcript TPM GCT).

### 2.3 Multi-factor scoring and machine-learning prioritization

Five biological criteria were z-score normalized and combined (Module 7): expression magnitude (20%), tumor specificity (30%), detection fraction (20%), immunogenicity (20%), and inverse escape liability (10%). Combined discovery score (expression-based; a discovery ranking, not a measure of CAR-T suitability) = weighted sum of z-scored features with optional +0.10 bonuses from beta-VAE and GCN modules when available.

Gradient-boosted models (Module 9) used LightGBM (num_leaves = 31, learning_rate = 0.01, 1,000 rounds, early stopping 50) and XGBoost (max_depth = 6, n_estimators = 100). SHAP TreeExplainer attributions are shown in Supplementary Figure S2. A stacked meta-learner (Module 13), beta-VAE (Module 15; latent = 24; final loss ∼2,986), and a three-layer GCN (Module 16; top 80 genes, Pearson r > 0.3, hidden = 64; final loss ∼240.4) provided exploratory network context (Supplementary Figure 3).

### 2.4 Curated surfaceome filter and CAR-T feasibility scoring

*Bona fide* CAR-T surface antigens were defined by scripts/utils/surfaceome_filter.py: plasma-membrane localization (UniProt), Extracellular domain (ECD) length ≥20 aa, and exclusion of ribosomal, nuclear, cytoplasmic, and secreted-only proteins. Of 83 scored genes, 13 passed as Tier A, and 70 were excluded (Table 1).

**TABLE 1:** Tier A *bona fide* plasma-membrane antigen catalog and CAR-T feasibility assessment (n = 13 of 83 input genes).

Gene	CAR-T feasibility label	Detection fraction (%)	Combined safety	Safety × coverage	HPA protein distribution	GTEx brain max TPM	GTEx peripheral max TPM	HPA blood MS (pg/L)	Relative gated rank (0–1)	Liability-laden rank	Topology class	Max ECD loop (aa)	Antibody tractable	Therapeutic index
*LIFR*	Antibody-tractable experimental priority	33.4	0.405	0.135	Detected in single	14.5	56.2	200,000.0	0.619	—	type_I_receptor	789	True	0.592
*SLC7A2*	Filtered (multi-pass transporter topology)	40.8	0.600	0.245	Detected in single	4.747	89.5	—	—	0.854	multi_pass_transporter	38	False	0.455
*VAMP5*	Filtered (tail-anchored/minimal surface exposure)	36.5	0.398	0.145	Detected in many	62.2	227.7	—	—	0.533	tail_anchored_SNARE	23	False	0.160
*SLC16A1*	Filtered (multi-pass transporter topology)	27.5	0.500	0.137	Detected in many	20.8	32.2	15,000.0	—	0.606	multi_pass_transporter	11	False	0.852
*CD9*	Filtered (ubiquitous normal-tissue protein)	53.2	0.231	0.123	Detected in all	123.4	227.4	4,200,000.0	—	0.554	multi_pass_transporter	84	False	0.234
*APP*	Filtered (systemic on-target risk)	50.9	0.225	0.115	Detected in many	561.1	324.9	86,000,000.0	—	—	type_I_receptor	684	True	0.157
*APMAP*	Filtered (ubiquitous normal-tissue protein)	30.6	0.253	0.078	Detected in all	323.9	252.5	110,000,000.0	—	—	type_I_receptor	355	True	0.121
*MCAM*	Filtered (ubiquitous normal-tissue protein)	27.3	0.232	0.063	Detected in all	121.9	398.9	200,000,000.0	—	—	type_I_receptor	536	True	0.068
*CD302*	Filtered (low tumor coverage)	18.2	0.329	0.060	Detected in many	6.237	62.3	95,000.0	—	—	type_I_receptor	146	True	0.292
*EVI2A*	Filtered (low tumor coverage)	8.400	0.662	0.056	Not detected	158.5	42.2	—	—	—	type_I_receptor	103	True	0.199
*BST2*	Filtered (ubiquitous normal-tissue protein)	26.3	0.197	0.052	Detected in all	52.1	350.5	140,000.0	—	0.378	multi_pass_transporter	113	False	0.075
*C7*	Filtered (ubiquitous normal-tissue protein)	20.3	0.223	0.045	Detected in all	3.648	355.7	18,000,000,000.0	—	—	type_I_receptor	821	True	0.057
*STING1*	Filtered (insufficient safety data)	32.6	—	—	—	—	—	—	—	0.243	multi_pass_transporter	10	False	—

Columns: Gene symbol; surfaceome tier; Gated nomination score (0–1, NaN if filtered); Combined safety (50% GTEx whole-body +50% HPA protein); Tumor-cell detection fraction (%); GTEx brain max TPM; GTEx critical peripheral max TPM; HPA protein tissue distribution; HPA MS blood (pg/L); Safety × coverage; CAR-T feasibility label. Antibody-tractable priority (n = 1): *LIFR* (gated relative rank 0.619, type I receptor, ECD loop 789 aa, safety 0.41, safety × coverage 0.135). Topology-filtered: *SLC7A2* (liability-laden rank 0.854, multi-pass transporter, max loop 38 aa, safety × coverage 0.245); *SLC16A1* (max loop 11 aa); *VAMP5* (tail-anchored). Filtered: *CD9* (ubiquitous, 53.2% detection fraction, MS blood 4.2 M pg/L); APP (systemic, brain 561.1 TPM); *MCAM*, *BST2*, *C7*, *APMAP* (ubiquitous); *CD302* (detection fraction 18.2% < 25%); *EVI2A* (detection fraction 8.4% < 25%); *STING1* (insufficient GTEx/HPA data). Row-level detail: *LIFR*: gated score 0.619, safety 0.41, detection fraction 33.4%, brain TPM 14.5 (antibody-tractable experimental priority) *SLC7A2*: gated score LL-rank 0.854, safety 0.60, detection fraction 40.8%, brain TPM 4.7 (filtered (multi-pass transporter topology)) *VAMP5*: gated score LL-rank 0.533, safety 0.40, detection fraction 36.5%, brain TPM 62.2 (filtered (tail-anchored/minimal surface exposure)) *SLC16A1*: gated score LL-rank 0.606, safety 0.50, detection fraction 27.5%, brain TPM 20.8 (filtered (multi-pass transporter topology)) *CD9*: gated score LL-rank 0.554, safety 0.23, detection fraction 53.2%, brain TPM 123.4 (filtered (ubiquitous normal-tissue protein)) *APP*: gated score filtered, safety 0.23, detection fraction 50.9%, brain TPM 561.1 (filtered (systemic on-target risk)) *APMAP*: gated score filtered, safety 0.25, detection fraction 30.6%, brain TPM 323.9 (filtered (ubiquitous normal-tissue protein)) *MCAM*: gated score filtered, safety 0.23, detection fraction 27.3%, brain TPM 121.9 (filtered (ubiquitous normal-tissue protein)) *CD302*: gated score filtered, safety 0.33, detection fraction 18.2%, brain TPM 6.2 (filtered (low tumor coverage)) *EVI2A*: gated score filtered, safety 0.66, detection fraction 8.4%, brain TPM 158.5 (filtered (low tumor coverage)) *BST2*: gated score LL-rank 0.378, safety 0.20, detection fraction 26.3%, brain TPM 52.1 (filtered (ubiquitous normal-tissue protein)) *C7*: gated score filtered, safety 0.22, detection fraction 20.3%, brain TPM 3.6 (filtered (ubiquitous normal-tissue protein)) *STING1*: filtered (insufficient safety data).

GTEx v8 normal-tissue safety used two layers on the same GENCODE v26 annotation. (i) Gene-level median TPM (Portal API medianGeneExpression/gene TPM GCT) underpins the primary atlas safety scores (whole-body safety = 0.30 × brain tier (max_brain_TPM) + 0.70 × peripheral tier (max_critical_TPM) across the 12 critical tissues listed above). (ii) For isoform-matched off-tumor risk, we downloaded the official GTEx v8 RSEM transcript TPM matrix (GTEx_Analysis_2017–06–05_v8_RSEMv1.3.0_transcript_tpm.gct.gz) and computed per-transcript median TPM within each SMTSD tissue. Tissue-wise medians for GTEx-present GENCODE v26 isoforms of narrative antigens were LIFR ENST00000263409.8 (brain-max median TPM 7.67, critical-max median TPM 27.88, transcript safety 0.26); SLC7A2 ENST00000398090.3 (brain-max median TPM 0.0, critical-max median TPM 31.22, transcript safety 0.44); CD9 ENST00000009180.8 (brain-max median TPM 148.9, critical-max median TPM 309.6, transcript safety 0.0); VAMP5 ENST00000306384.4 (brain-max median TPM 44.07, critical-max median TPM 141.9, transcript safety 0.06); SLC16A1 ENST00000369626.7 (brain-max median TPM 13.68, critical-max median TPM 22.235, transcript safety 0.26). Prior ENST pins for LIFR (ENST00000263433) and VAMP5 (ENST00000216268) mapped to PPP1R12C and ZBED4 under GENCODE v26 and were corrected to the GTEx-dominant isoforms of the intended genes; CD9 ENST00000382595 and SLC7A2 ENST00000412365 are absent from GENCODE v26 (Supplementary Table S4a; Supplementary Figure S10a). In parallel, all 15 GSE183655 FASTQ runs were re-quantified against GENCODE v26 with kallisto|bustools; after barcode transfer, 69,311 annotated cells were matched (17,162 meningioma/52,149 TME). On this common reference, priority-antigen tumor-versus-TME expression was: LIFR tumor/TME mean ratio 0.77 (tumor detection 38.4%, mean UMI 0.90); SLC7A2 tumor/TME mean ratio 1.61 (tumor detection 41.4%, mean UMI 1.95); CD9 tumor/TME mean ratio 1.55 (tumor detection 65.7%, mean UMI 7.15); VAMP5 tumor/TME mean ratio 0.93 (tumor detection 39.6%, mean UMI 1.34); SLC16A1 tumor/TME mean ratio 1.22 (tumor detection 31.6%, mean UMI 0.74) (Supplementary Figure S10b–d; Supplementary Table S4b). Deposited Cell Ranger gene-level tumor/TME statistics are retained for concordance (Supplementary Figure S10d).

Human Protein Atlas v25.1 JSON entries (proteinatlas.org/{ENSG}.json) provided immunohistochemistry (IHC) tissue distribution, MS blood concentration (pg/L), and subcellular localization. Combined safety = 0.50 × GTEx whole body +0.50 × HPA protein safety. Nomination weights: safety 50%, expression 25%, literature 10%, ECD accessibility 15%. The sensitivity of Tier A ranking to these hard-coded weights is quantified in Supplementary Figure S9 and Supplementary Table S5. Blood MS concentrations (e.g., CD9) cite the HPA field “Blood concentration–Conc. Blood MS [pg/L]” (Supplementary Material).

Membrane-topology gate: antibody-tractable = single-pass type I/II (contiguous ECD ≥80 aa) or GPI-anchored; poor-tractability = SLC/ABC transporters, tail-anchored SNAREs (VAMP family), multi-pass proteins with largest extracellular loop <80 aa. The largest loop length, not the summed ECD, defines epitope accessibility.

CAR-T quality gates: ubiquitous HPA protein; combined safety <0.35; detection fraction <25%; poor topology. Safety × coverage = combined safety × (detection fraction/100). Relative gated-rank score = min-max normalized 50% safety +25% expression +10% literature +15% ECD within Tier A survivors (not absolute feasibility). Stringent favorable: safety ≥0.55, detection fraction ≥50%, safety × coverage ≥0.30 (n = 0 in this cohort). LIFR is the only antibody-tractable experimental priority (gated rank 0.619; safety × coverage 0.135).

### 2.5 Cross-cohort expression reproducibility

The identical expression-scoring framework was applied to GSE136661, GSE16581, and GSE165341. Pairwise top-10 overlap: overlap% = 100 × |Top10_A n Top10_B|/10; Spearman rho on shared genes (n ∼ 83). Six comparisons yielded a mean overlap of 93.3% (range 90%–100%) and rho = 0.873–0.946 (all p < 0.001; Figure 3; Supplementary Table S2).

**FIGURE 3:**
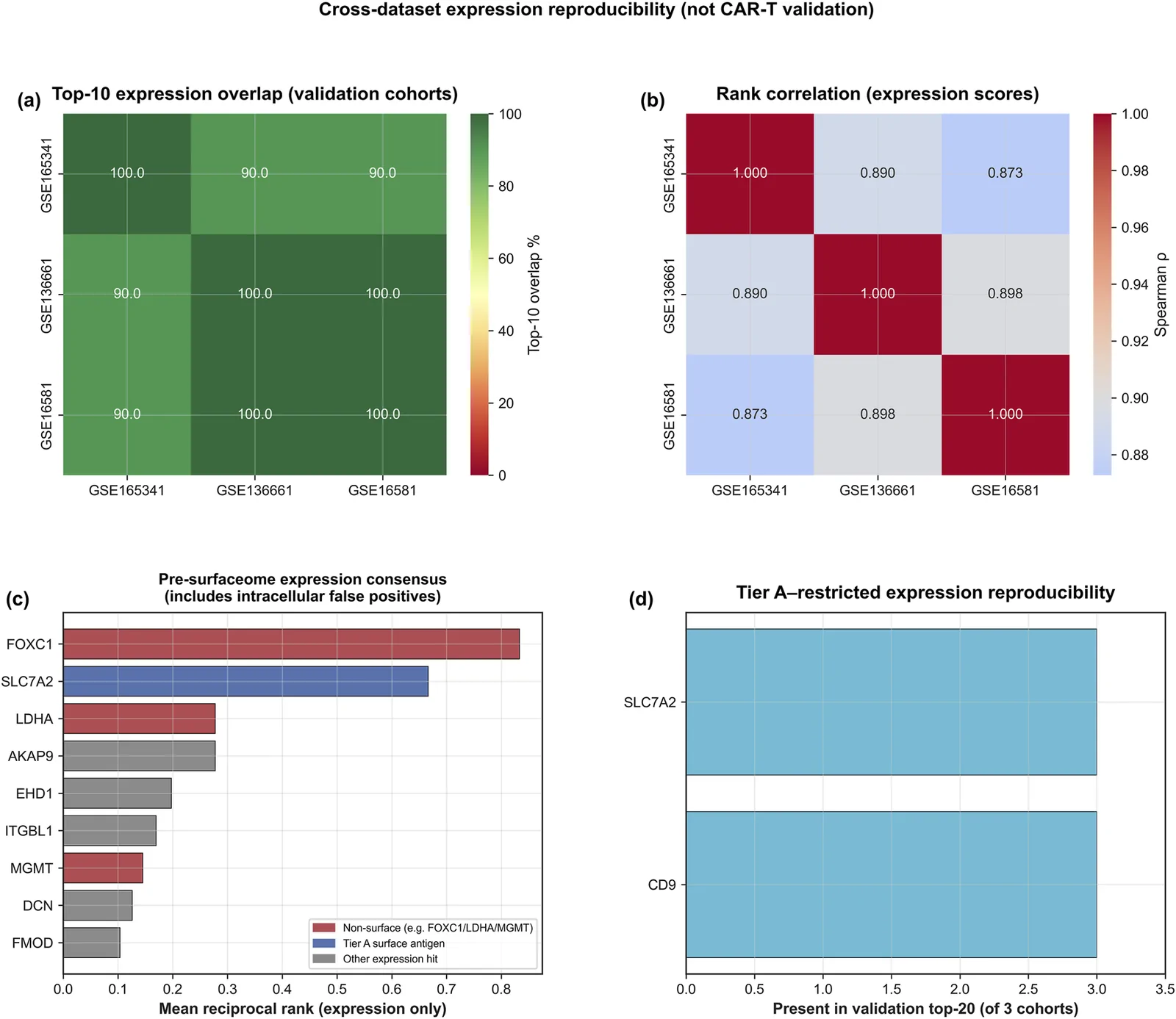
Cross-dataset expression reproducibility across independent meningioma cohorts (expression reproducibility only; not CAR-T validation). **(a)** Pairwise top-10 expression overlap heatmap among GSE165341, GSE136661, and GSE16581 (90%–100% shared top-10 genes). **(b)** Spearman rank-correlation heatmap (ρ = 0.873–0.898 across pairs). **(c)** Pre-surfaceome expression consensus: mean reciprocal rank for nine genes in all three validation top-10 lists; red = intracellular false positives (FOXC1, LDHA, and MGMT); blue = Tier A (SLC7A2); gray = other hits. Supplementary Table S6. **(d)** Tier A–restricted reproducibility: cohorts (of three) in which each Tier A gene appears in the top 20 genes expressed (SLC7A2 and CD9: 3/3). Supplementary Table S8. High overlap does not establish surface display or CAR-T suitability.

### 2.6 Structure-based docking and ECD accessibility (exploratory)

Extracellular domain boundaries were mapped from UniProt (Module 17). AlphaFold Database v6 models provided per-residue predicted local distance difference test (pLDDT) and solvent accessible surface area (SASA). The conformation score integrates ECD SASA, exposed fraction, mean ECD pLDDT, ECD length, epitope propensity, and glycosylation penalty (Supplementary Figure S7). SLC7A2 conformation score = 0.757; CD9 = 0.809; BST2 = 0.939.

HADDOCK3 v3 (Modules 18–21) docked epitope fragments with a generic anti-CD19 Fab surrogate (PDB 7JIC) under ambiguous distance restraints (2.0 A). Seven cohort antigens were screened as H-only and full Fab H + L; flexref scores are exploratory and excluded from nomination (Figure 4; Table 2; Supplementary Figures S6-7). OpenMM explicit-solvent MD (∼1 ns; +2 ns for selected antigens) followed by flexref. MM-GBSA GBn2 rank order is reported in Supplementary Table S3 (absolute ΔG values omitted due to force-field scaling).

**FIGURE 4:**
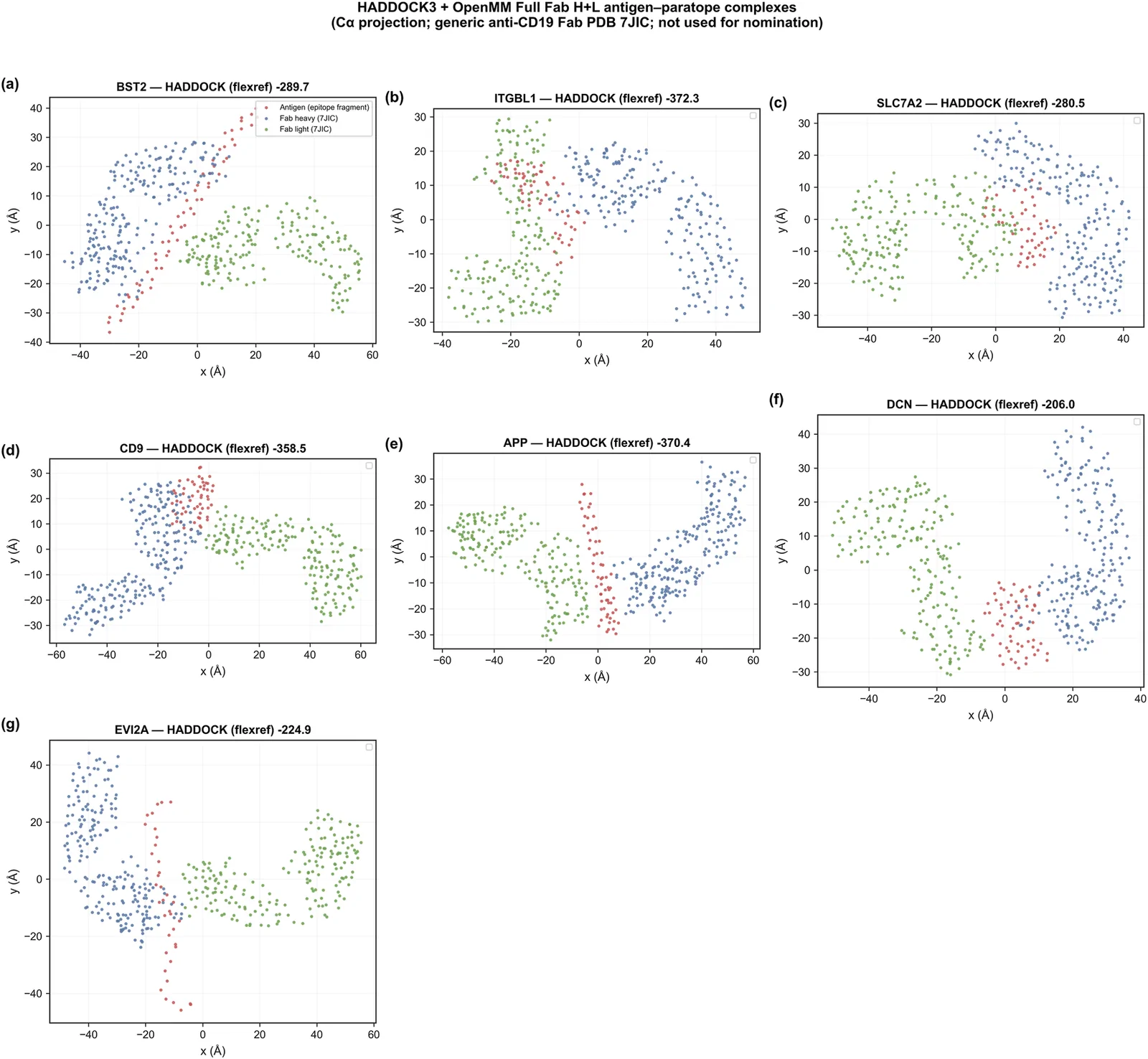
Exploratory HADDOCK3 + OpenMM Full Fab H + L structural models (seven-antigen cohort; generic anti-CD19 Fab PDB 7JIC; not used for nomination). Cα projections (red = antigen; blue = Fab heavy 7JIC; green = Fab light 7JIC). Flexref scores: **(a)** BST2 −289.7, **(b)** ITGBL1 −372.3, **(c)** SLC7A2 −280.5, **(d)** CD9 −358.5, **(e)** APP −370.4, **(f)** DCN −206.0, and **(g)** EVI2A −224.9. ITGBL1 and APP score most negatively despite Tier A exclusion, decoupling docking geometry from nomination. SLC7A2 priority derives from safety × coverage (0.245), not flexref. Mean full Fab improvement vs. H-only = −143.4 units. MM-GBSA GBn2 relative rank: EVI2A (Ostrom et al., 2023), BST2 (Goldbrunner et al., 2021), SLC7A2 (Louis et al., 2021), CD9 (June and Sadelain, 2018), DCN (O'Rourke et al., 2017), ITGBL1 (Brown et al., 2016), and APP (Majzner et al., 2022). See Table 2 and Supplementary Table S3.

**TABLE 2:** Exploratory HADDOCK3 + OpenMM flexref scores (seven-antigen cohort; generic PDB 7JIC Fab surrogate; not nomination evidence).

Gene	Tier a surface	H-only flexref (exploratory)	Full Fab H + L flexref (exploratory)	Δ (full Fab − H-only)	ECD conformation score	ECD length (aa)
*SLC7A2*	Yes	−161.0	−280.5	−119.5	0.757	127
*DCN*	No/excluded	−131.2	−206.0	−74.9	0.763	343
*ITGBL1*	No/excluded	−189.0	−372.3	−183.3	0.815	471
*CD9*	Yes	−162.6	−358.5	−196.0	0.809	106
*BST2*	Yes	−191.7	−289.7	−98.1	0.939	113
*APP*	Yes	−144.4	−370.4	−226.0	0.847	684
*EVI2A*	Yes	−119.0	−224.9	−105.8	0.845	103

Columns: Gene; H-only flexref; full Fab H + L flexref; Δ (full Fab minus H-only); Tier A status; CAR-T feasibility label. H-only scores: *ITGBL1* −189.0/−372.3; CD9 −162.6/−358.5; *APP* −144.4/−370.4; *SLC7A2* −161.0/−280.5; *BST2* −191.7/−289.7; *EVI2A*−119.0/−224.9; *DCN* −131.2/−206.0. Full Fab (more negative = more favorable geometry under restraints): *ITGBL1* −372.3 (excluded non-Tier-A Extracellular Matrix (ECM)); *APP* −370.4 (filtered, brain 561.1 TPM); *CD9* −358.5 (filtered ubiquitous); *BST2* −289.7 (filtered ubiquitous); *SLC7A2* −280.5 (experimental priority); *EVI2A* −224.9 (filtered low coverage); *DCN* −206.0 (excluded secreted ECM). Mean full Fab Δ vs. H-only: −143.4. Inference: *ITGBL1* and *APP* score the most negative yet are unsuitable CAR-T targets. Flexref does not predict clinical safety.

### 2.7 Statistical analysis, software, and reproducibility

Multiple-testing correction: Benjamini–Hochberg FDR at alpha = 0.05. Rank correlations: two-sided Spearman (SciPy). Software: Python 3.10+, Scanpy 1.9, pandas 2.x, NumPy, SciPy, scikit-learn, LightGBM 4.x, XGBoost 2.x, SHAP 0.4x, PyTorch 2.x, OpenMM 8.x, HADDOCK3 3.x, AlphaFold DB v6, GTEx Portal API v8, HPA v25.1, matplotlib/seaborn. Other details can be found in the code (see Data Availability Statement); extended derivations are in Supplementary Material.

## 3 Results

### 3.1 A two-stage framework separating transcriptomic discovery from CAR-T nomination

We developed a 24-module computational pipeline integrating patient-aware single-cell differential expression, curated surfaceome filtering, whole-body GTEx safety, Human Protein Atlas (HPA) protein cross-reference, and quantitative CAR-T feasibility gates (Figure 1). Applied to GSE183655 (80,220 QC-pass cells from eight patients), the workflow converts an initial list of 83 scored genes into a high-confidence catalog of 13 *bona fide* plasma-membrane antigens while filtering 70 transcriptomic false positives (Table 1). The framework is designed to be transferable to other CNS solid tumors and provides explicit safety × coverage criteria for single-antigen CAR-T nomination. Throughout, we distinguish discovery (expression-based ranking) from nomination (CAR-T suitability): a high discovery rank does not imply CAR-T suitability, which is established only by the surfaceome, systemic safety, and membrane-topology gates.

### 3.2 High-resolution single-cell atlas of meningioma

GSE183655 comprised 80,901 cell-called barcodes (Cell Ranger); 80,220 cells passed QC (99.2% retention; median 2,913 genes/cell; Supplementary Figure S1). Leiden clustering and curated marker panels resolved meningioma, immune, stromal, and vascular compartments (Figure 2). Patient-stratified differential expression identified 5,214 consistently upregulated genes (FDR <0.05, |log_2_FC| ≥ 1 in≥2/8 patients). An initial transmembrane-helix filter nominated 100 surface proteins but admitted numerous intracellular and secreted false positives (Supplementary Figure S4). Priority-antigen expression across these compartments is detailed in Supplementary Figure S8.

### 3.3 Curated surfaceome restriction recovers 13 Tier A plasma-membrane antigens

Curated surfaceome gating (UniProt plasma-membrane localization, ECD ≥20 aa, exclusion of ribosomal/nuclear/secreted genes) refined 83 candidates to 13 Tier A antigens (Figure 5). This step removed 70 former high-scoring genes, including FOXC1 (nuclear), lactate dehydrogenase A (LDHA) (cytoplasmic), MGMT (nuclear), and PTGDS (secreted; GTEx brain max = 2163 TPM). The resulting shortlist represents the first systematically filtered meningioma surfaceome resource from this cohort, suitable for flow cytometry, surface proteomics, and bispecific design.

**FIGURE 5:**
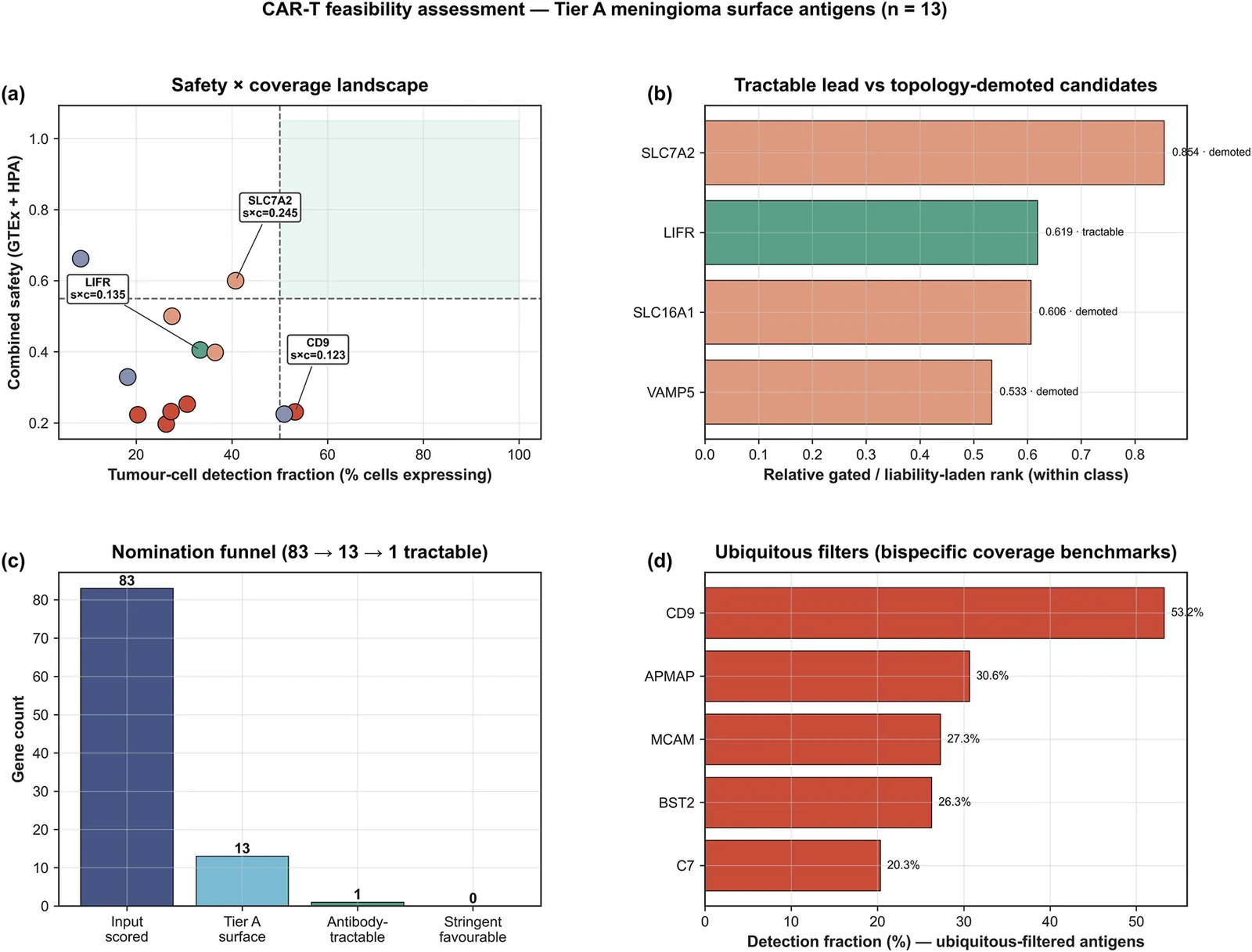
CAR-T feasibility and membrane-topology assessment (Tier A surface antigens, n = 13). **(a)** Safety × coverage landscape: combined safety (GTEx v8 + HPA v25.1, y-axis) versus tumor-cell detection fraction (x-axis). Dashed lines at safety = 0.55 and detection fraction = 50%; shaded quadrant = stringent favorable zone. No Tier A gene meets both thresholds. SLC7A2 (safety × coverage = 0.245; safety 0.60; 40.8%) is topology-filtered; LIFR (safety × coverage = 0.135; safety 0.41) is the only antibody-tractable priority. **(b)** Relative gated/liability-laden rank scores: LIFR 0.619 (antibody-tractable) versus SLC7A2 0.854, SLC16A1 0.606, and VAMP5 0.533 (topology-demoted). Topology gate: SLC7A2/SLC16A1 demoted (multi-pass transporter); VAMP5 (tail-anchored); LIFR retained (type I, 789-aa contiguous ECD). **(c)** Nomination funnel: 83 inputs scored → 13 Tier A → 1 antibody-tractable priority (LIFR) → 0 stringent favorable zone. **(d)** Ubiquitous-filter benchmarks: detection fraction for CD9 (53.2%), APMAP (30.6%), MCAM (27.3%), BST2 (26.3%), and C7 (20.3%). Weight sensitivity: Supplementary Figure S9 and Supplementary Table S5.

### 3.4 Whole-body GTEx and HPA protein data strengthen safety nomination

Systemic CAR-T safety integrated GTEx v8 whole-body transcriptomics (30% brain, 70% critical peripheral tissues) with HPA protein-level evidence (IHC tissue distribution, MS blood concentration) in a 50:50 combined safety score (50% weight in nomination; Supplementary Figure S5). Exact Ensembl ID matching corrected prior mapping artefacts. 5 Tier A genes with HPA protein “Detected in all tissues” were filtered from ranking (*CD9*, *MCAM*, *BST2*, *C7*, and *APMAP*), including CD9 (Human Protein Atlas field “Blood concentration–Conc. Blood MS [pg/L]” ≈ 4.2 million pg/L; HPA mass-spectrometry plasma estimate, not an in-house assay), a platelet/leukocyte tetraspanin with 53.2% tumor-cell detection fraction but combined safety 0.23. Amyloid Precursor protein (APP) (GTEx brain max = 561.1 TPM) was filtered for high CNS expression. These filters prevent confident but unsafe nomination of ubiquitous or CNS-abundant antigens.

### 3.5 Membrane-topology tractability filtering refines experimental priorities

A membrane-topology tractability gate was applied after surfaceome and safety filtering (Figure 3; Table 1). Antibody-tractable classes: single-pass type I/II receptors and GPI-anchored proteins with contiguous extracellular domains; poor-tractability classes: multi-pass transporters (SLC family), tail-anchored SNAREs, and multi-pass proteins with extracellular loops <80 aa. Of 13 Tier A antigens, seven are topologically antibody-tractable; three were demoted, including SLC7A2 (14-TM cationic amino-acid transporter; largest loop 38 aa, sum ECD 127 aa), SLC16A1 (largest loop 11 aa), and VAMP5 (tail-anchored SNARE; ECD 23 aa).

Under stringent nomination thresholds (combined safety ≥0.55, detection fraction ≥50%, safety × coverage ≥0.30), n = 0 Tier A antigens qualify. SLC7A2 had the highest liability-laden relative rank (0.854) among topology-filtered genes, reflecting the best safety × coverage among transporters (combined safety 0.60, detection fraction 40.8%, safety × coverage 0.245), but is the least compromised, not favorable, and is a poor antibody target. The only antibody-tractable nominee passing safety/detection-fraction gates is LIFR (type I receptor; contiguous ECD 789 aa; gated relative rank 0.619; combined safety 0.41; safety × coverage 0.135), which remains liability-laden. Bispecific and logic-gated strategies are warranted (e.g., pairing coverage benchmark CD9, 53.2% detection fraction).

### 3.6 Cross-dataset analysis establishes expression reproducibility, not clinical validation

Independent replication across GSE136661, GSE16581, and GSE165341 yielded 93.3% mean top-ten overlap and Spearman rho = 0.873–0.946 (all p < 0.001; Figure 4; Supplementary Table S2), indicating that the mRNA-based ranking is reproducible across platforms. This reflects expression reproducibility, not validation of surface-protein display, CAR-T suitability, or clinical performance, and supports the atlas as a stable discovery resource, while CAR-T suitability is established only by the surfaceome, safety, and topology gates. Pre-surfaceome consensus genes (including intracellular false positives) and Tier A-restricted frequencies are listed in Supplementary Tables S6 and S8, corresponding to Figure 4 panels C–D.

### 3.7 Exploratory structural modeling supports epitope accessibility assessment

AlphaFold-guided ECD accessibility scoring quantified extracellular exposure for Tier A antigens (Supplementary Figure S7). HADDOCK3 + OpenMM docking with a generic anti-CD19 Fab (PDB 7JIC) was exploratory only (Figure 5; Table 2): SLC7A2 full Fab flexref = −280.5, CD9 = −358.5, APP = −370.4. Generic Fab scores reflect geometric contact under restraints and are not used for nomination.

### 3.8 Synthesis: a transferable nomination framework and tiered experimental priorities

This study delivers two linked resources for meningioma CAR-T discovery (Ostrom et al., 2023): a curated surfaceome atlas of 13 plasma-membrane antigens from GSE183655 and (Goldbrunner et al., 2021) a multi-layer nomination framework with explicit quality gates (surfaceome, GTEx/HPA systemic safety, membrane topology, safety × coverage) and a relative gated-rank score that must not be interpreted as absolute feasibility. Applied to this cohort, the gates produce tiered priorities: LIFR as the only antibody-tractable experimental lead (safety × coverage 0.135); SLC7A2 as the least-compromised on transcriptomic safety among transporters (safety × coverage 0.245) but topology-filtered. After topology filtering, LIFR is the only antibody-tractable Tier A nominee (safety × coverage 0.135, combined safety 0.41) but remains liability-laden under stringent thresholds; bispecific/logic-gated approaches are warranted. Recommended validation: flow cytometry/IHC for LIFR surface expression; surface proteomics; bispecific designs using CD9 (53.2% detection fraction) as a coverage benchmark paired with safer antigens.

## 4 Discussion

This study provides two linked resources for meningioma CAR-T discovery, a curated atlas of 13 *bona fide* plasma-membrane antigens and a transferable, multi-layer nomination framework, together with a cautionary demonstration of how naive *in silico* pipelines mislead. In our own initial analysis, a permissive transmembrane-helix filter admitted numerous non-surface false positives (ribosomal proteins, the nuclear factors FOXC1 and MGMT, the cytoplasmic enzyme LDHA, and secreted proteins such as PTGDS), and a brain-only safety screen mis-scored ubiquitously expressed antigens as safe. Correcting these errors changed the conclusions substantially, underscoring that the quality of the filters, not the elaborateness of the pipeline, determines whether a transcriptomic screen yields credible CAR-T targets.

Curated surfaceome restriction was the essential first gate. Requiring UniProt-supported plasma-membrane localization with a contiguous extracellular domain and excluding ribosomal, nuclear, cytoplasmic, and secreted proteins removed 70 of 83 candidates, including several that had ranked highly on tumor-enriched mRNA alone. This is consistent with the broader principle that transcript enrichment is necessary but not sufficient to identify a surface antigen, and that experimentally grounded surfaceome catalogs should bound any such analysis (Bausch-Fluck et al., 2015; Bausch-Fluck et al., 2018).

Systemic-safety scoring was equally decisive. Because CAR-T cells act throughout the body, we integrated whole-body GTEx transcriptomics with Human Protein Atlas protein-level evidence rather than relying on brain expression alone and weighted safety heavily in the nomination. This correctly demoted CD9, a tetraspanin with 53.2% tumor-cell coverage but pan-tissue protein expression and high plasma abundance (platelets and leukocytes), and other ubiquitously expressed antigens. Shared normal-tissue expression is the dominant cause of severe on-target off-tumor toxicity in solid-tumor CAR-T (Morgan et al., 2010; Lamers et al., 2013), and an antigen’s attractiveness on coverage cannot offset a poor normal-tissue profile.

A third gate, membrane-topology tractability, proved pivotal and is frequently omitted from *in silico* target nominations. Antibody and CAR binding require an accessible, contiguous ectodomain; multi-pass transporters and tail-anchored proteins expose only short extracellular loops and are poor antibody targets despite passing a length-based surfaceome filter. Applying a topology gate (single-pass type I/II or GPI-anchored with a contiguous ectodomain ≥80 aa classed as tractable) demoted the highest transcriptomic-safety transporter SLC7A2 (a 14-transmembrane cationic amino-acid transporter whose largest extracellular loop is only 38 aa), the transporter SLC16A1, and the tail-anchored SNARE VAMP5. The only antibody-tractable nominee that passed the safety and coverage gates was LIFR, a single-pass type I receptor with a contiguous 789-aa ectodomain. However, LIFR remained liability-laden, with only moderate systemic safety (0.41) and low safety × coverage (0.135).

The central finding follows directly: under stringent thresholds (combined safety ≥0.55, tumor-cell detection fraction ≥50%, safety × coverage ≥0.30), no Tier A antigen qualified. The leading candidates are least-compromised rather than favorable; SLC7A2 has the best transcriptomic safety among transporters but is topologically intractable, and LIFR is tractable but only moderately safe with low coverage. We regard this negative result as the study’s most useful contribution: in this cohort, meningioma lacks an ideal single CAR-T surface antigen. Explicit gates make that conclusion visible rather than obscuring it behind a confident but unsafe or intractable nomination. The practical implication is to pursue multi-antigen approaches; bispecific or tandem CARs to mitigate escape, and logic-gated circuits to spare normal tissue, potentially pairing a high-coverage but unsafe antigen such as CD9 with a safer partner under combinatorial control (Hegde et al., 2016; Roybal et al., 2016; Cho et al., 2018).

These results also clarify what cross-platform reproducibility and structural modeling can and cannot establish here. The high cross-dataset overlap of expression rankings (93.3%) demonstrates that the mRNA-based ranking is stable across cohorts. However, expression ranking stability is not external validation of CAR-T suitability; consistently expressed transcripts such as FOXC1, LDHA, and MGMT are reproducible but are not surface antigens. Likewise, docking each candidate against a generic, non-cognate anti-CD19 Fab measures geometric contact under restraints, not bindability, and we therefore excluded these scores from nomination and report them as exploratory only.

Finally, the framework is transferable. The same gates, surfaceome localization, whole-body protein-level safety, membrane-topology tractability, and safety × coverage apply to target nomination in other CNS and solid tumors, where the same failure modes recur. We present the atlas and gates as a reproducible scaffold for hypothesis generation, explicitly subordinate to experimental validation.

### 4.1 Limitations

All findings in this study are transcriptomic and computational; none has been confirmed at the protein or cell-surface level. Transcript abundance does not establish surface-protein display, and no flow cytometry, immunohistochemistry, or surface proteomics was performed. Every antigen on the atlas, including LIFR, is therefore a hypothesis, not a validated or trial-ready target, and each nomination requires surface-level confirmation on tumor and matched normal tissue before any therapeutic interpretation. A first-order limitation is that safety scoring remains allogeneic and bulk-derived: meningioma expression comes from GSE183655 patients, whereas normal-tissue references come from unrelated GTEx and Human Protein Atlas donors, not patient-matched normal meninges or brain. Gate thresholds, while biologically motivated, are not clinically calibrated. Extracellular-domain accessibility was inferred from AlphaFold models, which carry uncertainty for membrane proteins and do not capture glycosylation, and generic-antibody docking does not assess bindability. The discovery cohort comprises eight patients with the selection inherent to a single dataset, and the relative gated-rank score is a within-tier ranking, not an absolute feasibility probability. The machine-learning modules added limited incremental value beyond the multi-factor score. No functional validation (antigen-specific cytotoxicity) was undertaken. To address reference mismatch with GTEx v8, we completed isoform-resolved GTEx transcript analyses on GENCODE v26 (Supplementary Table S4a) and cohort-wide FASTQ re-quantification of all 15 GSE183655 runs against the same annotation with kallisto|bustools (Supplementary Figure S10; Supplementary Table S4b); primary discovery and nomination gates remain based on the deposited Cell Ranger gene-level atlas, with GENCODE v26 re-quant used for reference-matched tumor/TME reporting of priority antigens. Marker-based tumor calls lack CNV confirmation (Supplementary Material). Weight sensitivity analyses (Supplementary Figure S9; Supplementary Table S5) show rank stability but also that SLC7A2 ranks highly before topology gating.

### 4.2 Future directions

The immediate priority is experimental validation of surface-protein expression: flow cytometry and immunohistochemistry for LIFR and any other tractable candidate on primary meningioma and matched normal tissues (including liver and kidney for transporters and platelets and leukocytes for CD9), complemented by surface proteomics to confirm membrane localization and abundance. Given that no single antigen met stringent criteria, multi-antigen engineering is the logical path, tandem or bispecific CARs to counter antigen-negative escape, and logic-gated (e.g., synNotch or combinatorial) circuits to pair a high-coverage antigen such as CD9 with a safer partner and restore a therapeutic window. Larger, molecularly annotated cohorts integrating spatial and protein-level data would strengthen both discovery and safety estimation, and prospective evaluation should focus on recurrent and high-grade meningioma, where the clinical need is greatest. Finally, the gate-based nomination framework, surfaceome localization, whole-body protein-level safety, membrane-topology tractability, and safety × coverage can be applied to target discovery in other CNS and solid tumors as a guard against confident but non-viable nominations. With GENCODE v26–matched gene-level re-quantification now complete, further work can prioritize isoform-resolved single-cell quantification (where chemistry and depth allow) and, critically, protein-level validation on primary meningioma and matched normal tissues.

## 5 Conclusion

Rigorous surfaceome, whole-body safety, and membrane-topology gating of meningioma single-cell transcriptomes yields a reproducible 13-antigen surface atlas but identifies no single CAR-T target that meets stringent safety, coverage, and tractability criteria. LIFR is the only antibody-tractable lead and remains liability-laden. By exposing confident false positives that naive pipelines would nominate, the transferable gate framework argues for bispecific or logic-gated CAR-T strategies in meningioma rather than single-antigen designs.

## Data Availability

Publicly available datasets were analyzed in this study. These data can be found here: GSE183655: https://www.ncbi.nlm.nih.gov/geo/query/acc.cgi?acc=GSE183655; GSE136661: https://www.ncbi.nlm.nih.gov/geo/query/acc.cgi?acc=GSE136661; GSE16581: https://www.ncbi.nlm.nih.gov/geo/query/acc.cgi?acc=GSE16581; GSE165341: https://www.ncbi.nlm.nih.gov/geo/query/acc.cgi?acc=GSE165341.
