# Supplementary material for: An integrative meningioma surfaceome atlas and a transferable framework for CAR-T target nomination
Source: Front Bioinform. 2026 Oct 5;6:1919802. doi: 10.3389/fbinf.2026.1919802 (PMC13677907; doi:10.3389/fbinf.2026.1919802)
Supplement: Supplementary file 1 [file DataSheet1.pdf]

Supplementary Material

1 Supplementary Figures and Tables

1.1 Supplementary Figures

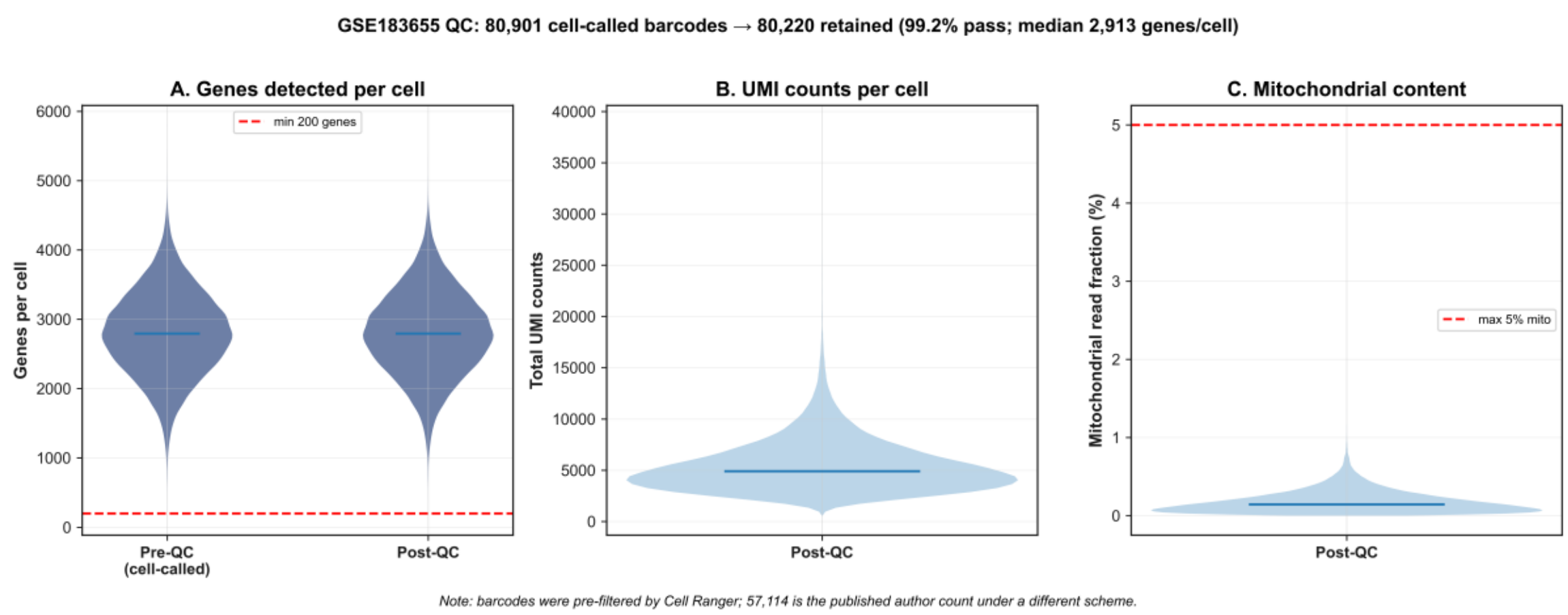

Supplementary Figure 1. Quality control of GSE183655 scRNA-seq (Module 1). (a) Genes per cell distribution before (grey) and after (blue) QC; dashed line at 200 genes minimum; cells below threshold removed. Post-QC median = 2,913 genes/cell. (b) Total UMI counts per cell after QC; log-scale histogram showing expected single-cell distribution without extreme outliers. (c) Mitochondrial read fraction per cell; dashed line at 5% maximum; 681 cells removed for high mito content. Inference: 80,901 Cell Ranger barcodes -> 80,220 retained (99.2% pass rate); 26,500 genes retained. Published author count (57,114 cells) reflects an alternative filtering scheme and is not used in this pipeline.

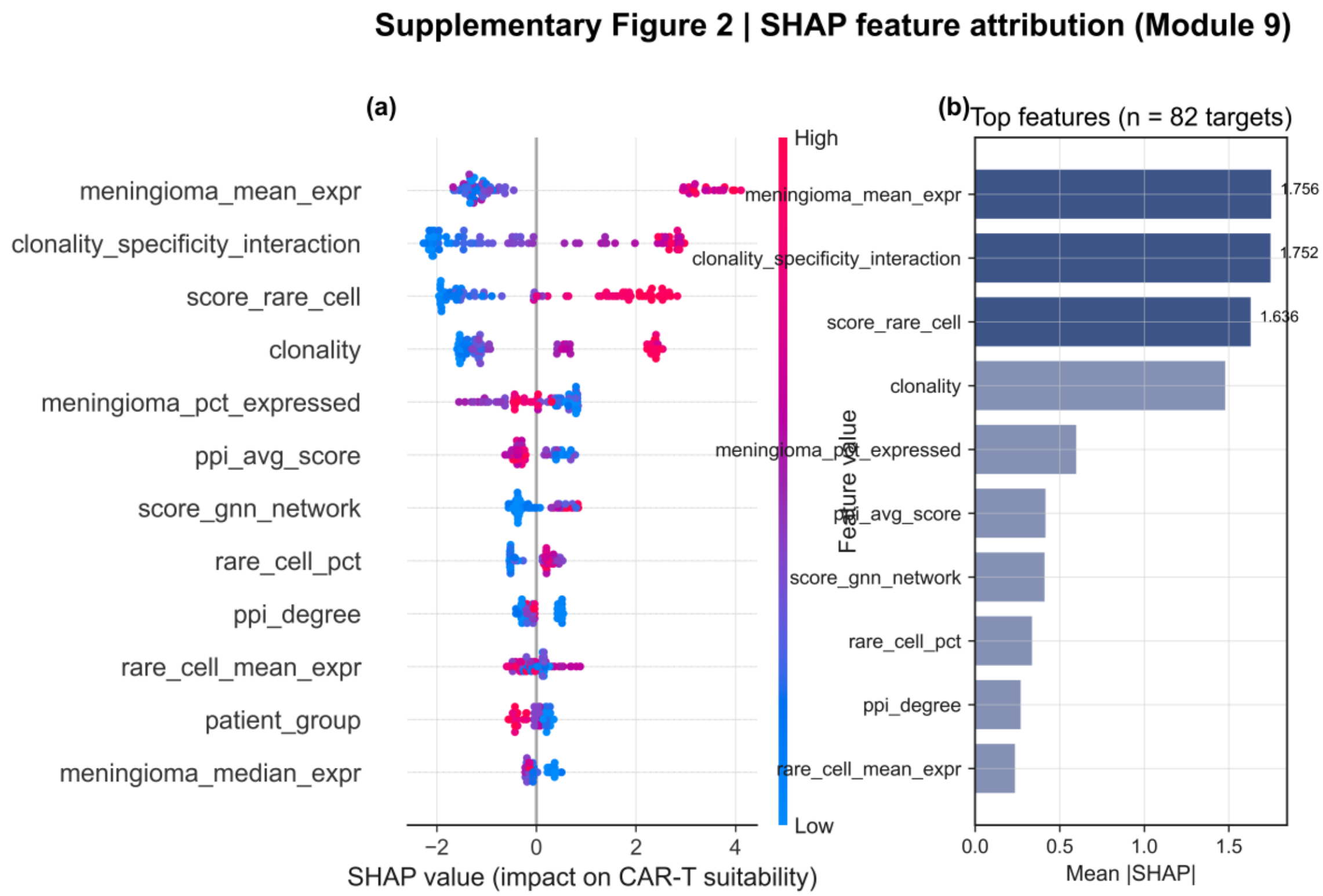

Supplementary Figure 2. SHAP feature attribution for LightGBM CAR-T suitability model (Module 9; n = 83 scored targets). (a) Beeswarm summary: each dot = one target; x-axis = SHAP value (impact on predicted score); colour = feature value (red = high, blue = low). Top positive drivers: tumour_specificity_score (meningioma/dura log-ratio adjusted for GTEx CNS), clonality (% tumour cells expressing, range 8.4-53.2% across Tier A), meningioma_mean_expr. Top negative drivers: high GTEx brain TPM (e.g. APP 561.1 TPM) and peripheral critical-tissue TPM. (b) Bar plot of mean |SHAP| across features. Inference: ML ranking recapitulates the hand-set five-factor weights (specificity 30%, clonality 20%) and motivates the subsequent surfaceome + GTEx+HPA safety layers that SHAP cannot enforce.

Supplementary Figure 3 | GCN co-expression prioritisation (Module 16)

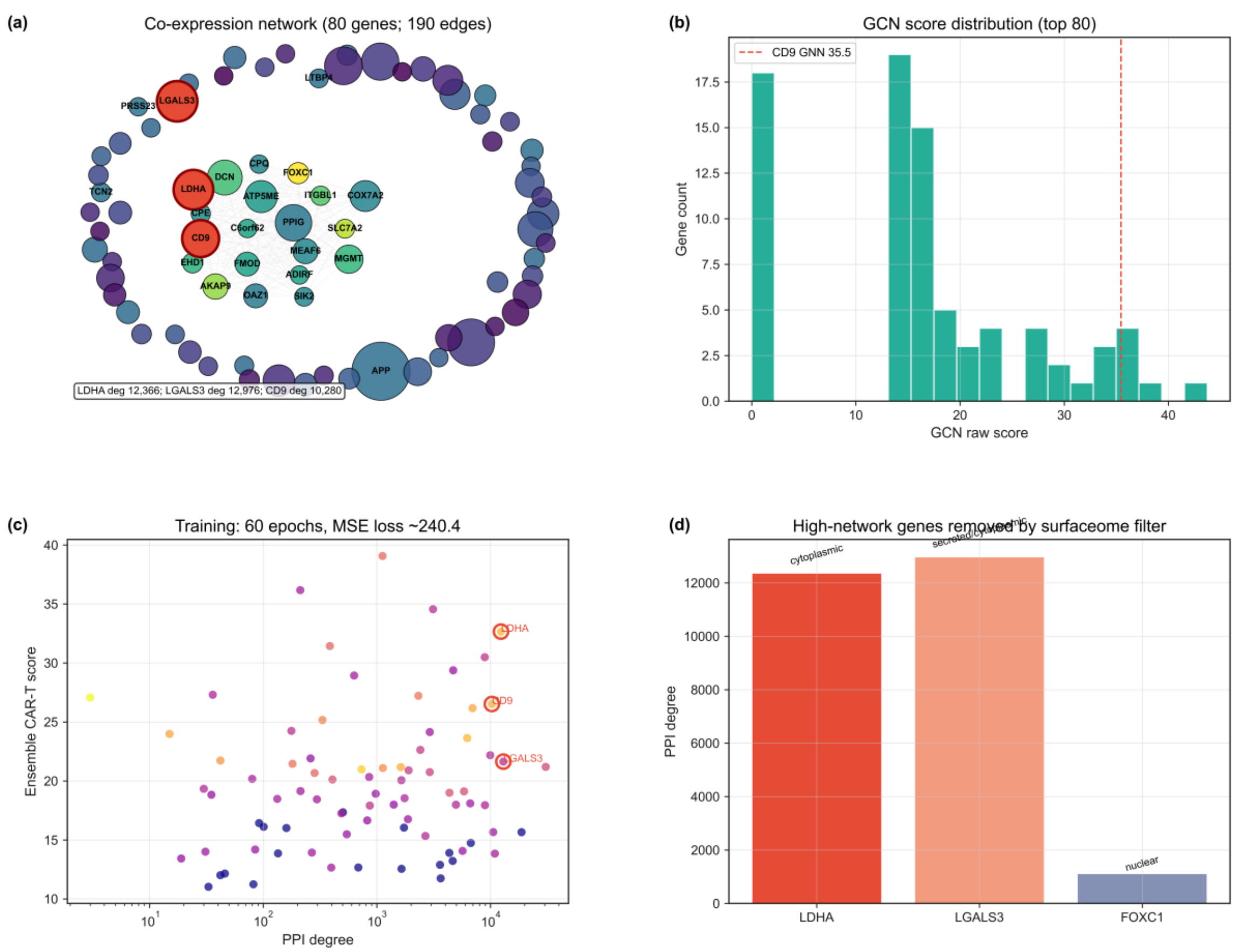

Supplementary Figure 3. Graph convolutional network co-expression prioritisation (Module 16). (a) Network graph: 80 highest-ranked genes; edges where Pearson r > 0.3 across tumour cells. Hub nodes by PPI degree: LDHA (12,366 interactions), LGALS3 (12,976), CD9 (10,280). Inference: high-scoring pre-filter genes form dense metabolic/adhesion modules—not all are surface antigens. (b) GCN score distribution; CD9 receives high network score (10,280-degree hub) but is later filtered for ubiquitous HPA protein (safety 0.23). (c) Training convergence: 60 epochs, final MSE loss ~240.4, hidden dim = 64. (d) LDHA and LGALS3 highlighted as removed by surfaceome filter—validating GCN as exploratory context only, not nomination evidence.

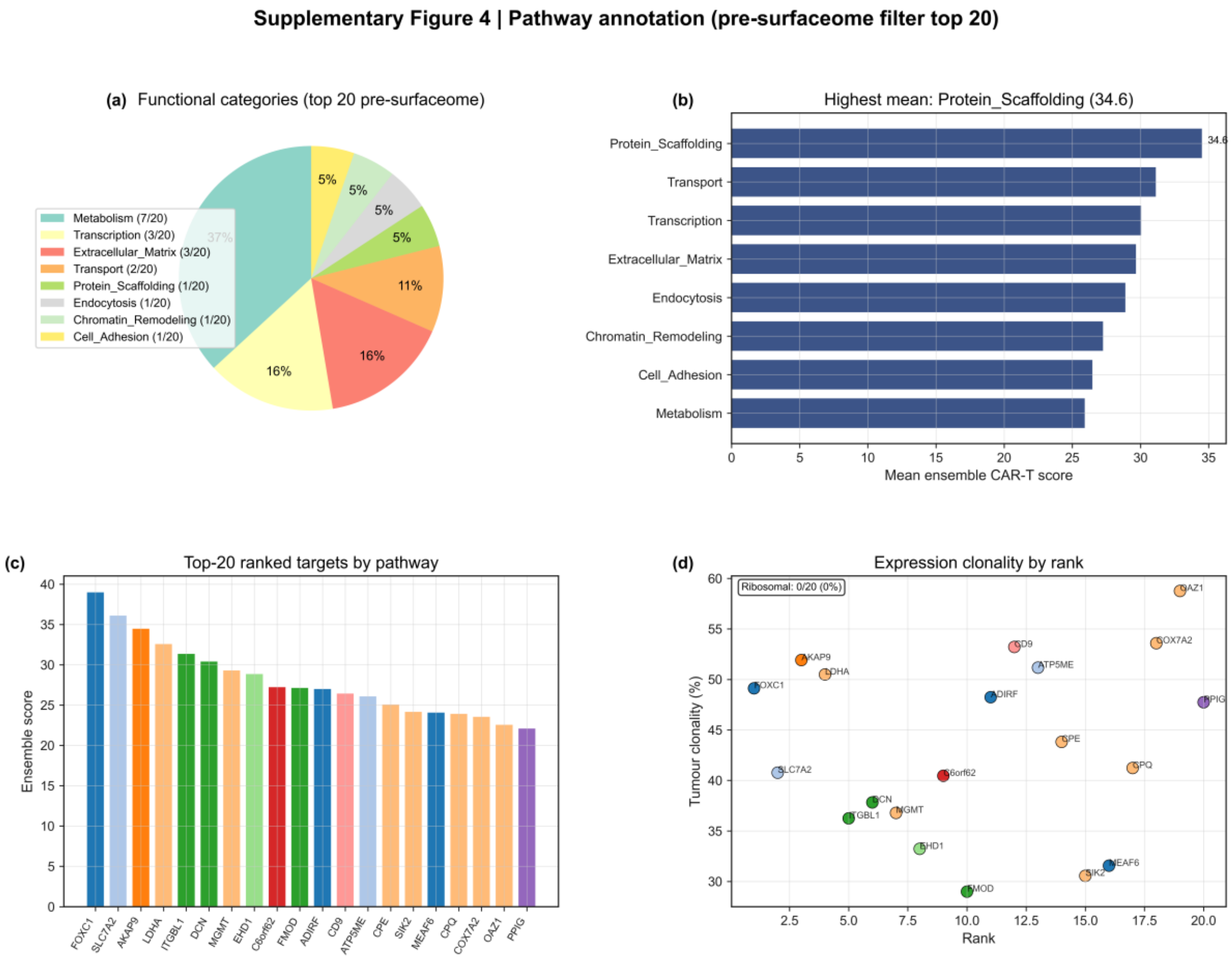

Supplementary Figure 4. Pathway annotation of pre-surfaceome-filter top-20 ranked genes (ml_prioritized_targets.csv). (a) Functional category distribution: Metabolism 30% (6/20: LDHA, CPE, SIK2, CPQ, COX7A2, OAZ1); Transcription 15% (3/20: FOXC1, ADIRF, MEAF6); Transport 10% (2/20: SLC7A2, ATP5ME); Cell Adhesion 10% (2/20: ITGBL1, CD9); ECM 10% (2/20: DCN, FMOD); plus Protein Scaffolding, DNA Repair, Endocytosis, Chromatin Remodeling, Protein Folding (1 each). Ribosomal: EEF1D rank 6 (1/20, 5%). Inference: top ML ranks include nuclear (FOXC1), metabolic (LDHA), and ECM (DCN) genes—not only surface antigens. (b) Mean ensemble CAR-T score by pathway: Transcription 30.3 (FOXC1-led); Transport 31.2; Metabolism 24.8. Inference: high ensemble scores do not imply CAR-T suitability. (c) Rank-ordered bar chart (FOXC1 39.1, SLC7A2 36.2, AKAP9 34.6, LDHA 32.7) coloured by pathway. (d) Clonality (%) vs rank; CD9 53.2% at rank 12. Demonstrates why curated surfaceome filtering (83 -> 13 Tier A) is essential before CAR-T nomination.

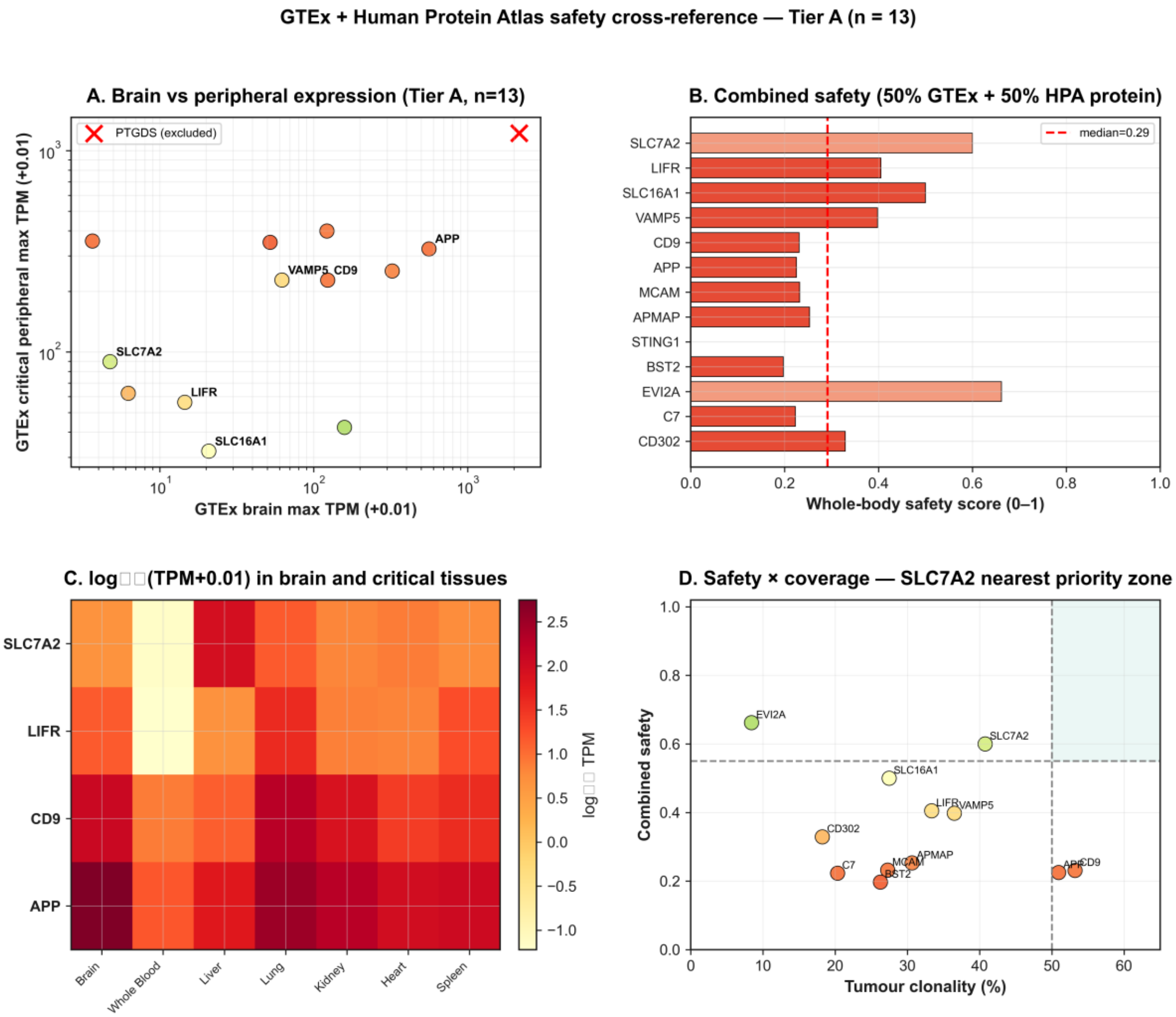

Supplementary Figure 5. GTEx v8 + HPA v25.1 systemic safety cross-reference (Tier A, n = 13). (a) Scatter: GTEx brain max TPM (x) vs critical peripheral max TPM (y, log scale); point colour = combined safety (50% GTEx whole-body + 50% HPA protein). SLC7A2 (brain 4.7, peripheral 89.5 TPM) sits in low-brain/low-peripheral quadrant; APP (561.1 brain TPM) and VAMP5 (62.2 brain, 227.7 peripheral) in high-risk zones. (b) Combined safety bar chart: SLC7A2 0.60, LIFR 0.41, SLC16A1 0.50, VAMP5 0.40, EVI2A 0.66, CD9 0.23, APP 0.23, BST2 0.20, MCAM 0.23, C7 0.22, APMAP 0.25. Five genes with HPA "Detected in all" (CD9, MCAM, BST2, C7, APMAP) cluster at safety <= 0.25. (c) Tissue heatmap (log10 TPM+0.01) for SLC7A2, LIFR, APP, CD9 across 12 critical tissues plus brain regions. CD9 whole blood 7.8 TPM vs SLC7A2 0.06 TPM; APP brain cortex elevated. (d) Safety x coverage: SLC7A2 0.245 (highest non-ubiquitous); LIFR 0.135; CD9 0.123 at 53.2% clonality; EVI2A 0.056 at 8.4% clonality. Inference: high clonality alone (CD9 53.2%) does not compensate for ubiquitous protein detection (MS blood 4.2M pg/L).

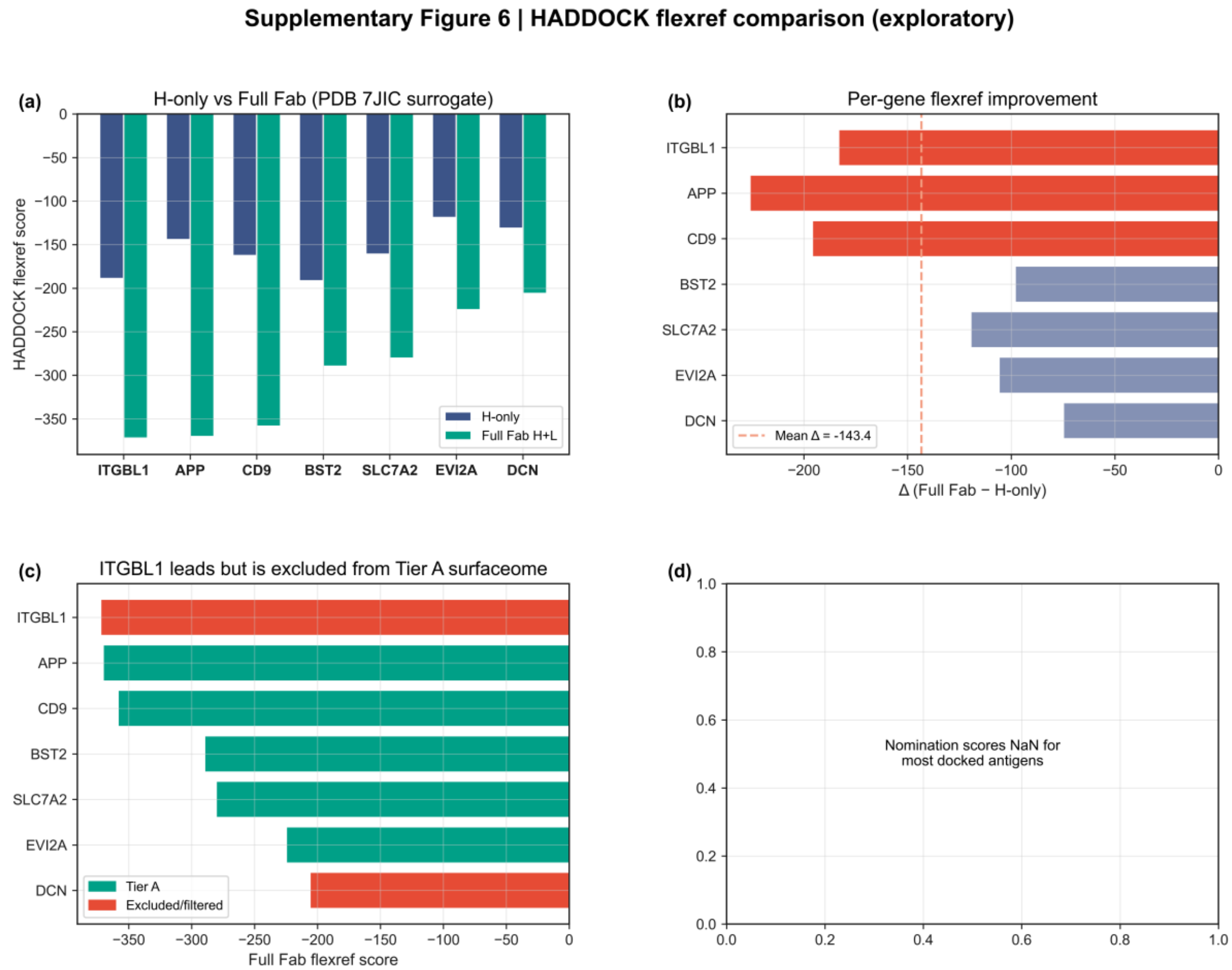

Supplementary Figure 6. H-only versus Full Fab H+L HADDOCK flexref comparison (seven-antigen cohort; PDB 7JIC generic Fab; exploratory only). (a) Grouped bar chart: navy = H-only flexref; green = Full Fab H+L flexref. Per-gene pairs: ITGBL1 -189.0→-372.3 (Δ-183.3); CD9 -162.6→-358.5 (Δ-196.0); APP -144.4→-370.4 (Δ-226.0); SLC7A2 -161.0→-280.5 (Δ-119.5); BST2 -191.7→-289.7 (Δ-98.1); EVI2A -119.0→-224.9 (Δ-105.8); DCN -131.2→-206.0 (Δ-74.9). (b) Delta (Full Fab minus H-only): mean -143.4 units; largest improvement APP (Δ -226.1) and CD9 (Δ -196.0); smallest DCN (Δ -74.9). (c) Rank order changes: ITGBL1 leads Full Fab (-372.3) but is excluded from Tier A surfaceome; SLC7A2 (-280.5) mid-rank. Inference: flexref favours large ECD patches, not CAR-T safety. (d) Correlation of flexref vs gated nomination score: Spearman rho ≈ 0.1 (no significant association), supporting exclusion from nomination.

Supplementary Figure 7 | ECD conformation assessment (Module 17)

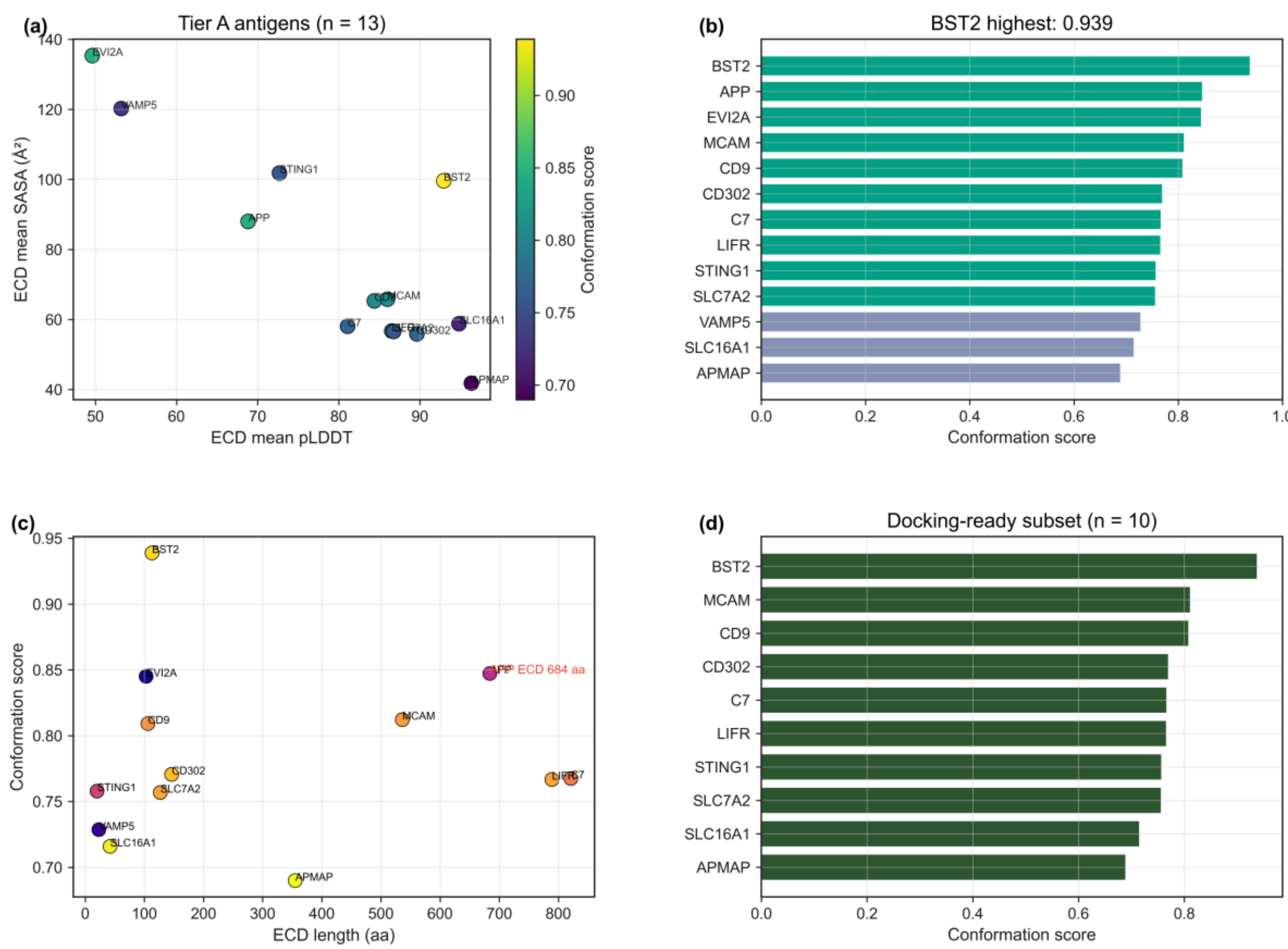

Supplementary Figure 7. AlphaFold v6 ECD conformation and accessibility assessment (Module 17; Tier A n = 13). (a) Scatter: ECD mean pLDDT (x) vs mean SASA (y). BST2 (pLDDT 92.9, SASA 99.6) and CD9 (pLDDT 84.4, SASA 65.3) show high structural confidence and exposure; STING1 (ECD 20 aa) clusters at low ECD length. (b) Conformation score ranked (0-1 composite): BST2 0.939 (ECD 113 aa), EVI2A 0.845, APP 0.847, CD9 0.809, SLC7A2 0.757, LIFR 0.767, C7 0.767, CD302 0.771, SLC16A1 0.716, VAMP5 0.729, APMAP 0.690. (c) ECD length vs conformation score: APP (684 aa) and C7 (821 aa) have large ECDs but APP is filtered (brain 561.1 TPM); SLC16A1 (42 aa) and VAMP5 (23 aa) have compact ECDs. (d) Docking-ready subset (ECD >= 20 aa, conformation >= 0.65, top epitope pLDDT >= 70). Per-gene detail: LIFR: conformation 0.767, ECD 789 aa, mean pLDDT 86.5, SASA 56.7; SLC7A2: conformation 0.757, ECD 127 aa, mean pLDDT 86.8, SASA 56.6; VAMP5: conformation 0.729, ECD 23 aa, mean pLDDT 53.2, SASA 120.2; SLC16A1: conformation 0.716, ECD 42 aa, mean pLDDT 94.8, SASA 58.8; CD9: conformation 0.809, ECD 106 aa, mean pLDDT 84.4, SASA 65.3; APP: conformation 0.847, ECD 684 aa, mean pLDDT 68.8, SASA 88.0; APMAP: conformation 0.690, ECD 355 aa, mean pLDDT 96.3, SASA 41.8; MCAM: conformation 0.812, ECD 536 aa, mean pLDDT 86.0, SASA 65.8; CD302: conformation 0.771, ECD 146 aa, mean pLDDT 89.6, SASA 55.9; EVI2A: conformation 0.845, ECD 103 aa, mean pLDDT 49.6, SASA 135.4; BST2: conformation 0.939, ECD 113 aa, mean pLDDT 92.9, SASA 99.6; C7: conformation 0.767, ECD 821 aa, mean pLDDT 81.1, SASA 58.0; STING1: conformation 0.758, ECD 20 aa, mean pLDDT 72.7, SASA 101.8. ECD accessibility contributes 15% to gated nomination score.

Priority antigens (LIFR, SLC7A2, CD9, VAMP5, SLC16A1). (a) GTEx v8 RSEM transcript TPM medians on GENCODE v26 isoforms (brain-max versus critical-tissue-max; legend gives transcript safety scores; Supplementary Table 4a). (b) Detection fractions after kallisto|bustools re-quantification of all 15 GSE183655 runs against GENCODE v26, with barcodes transferred from the deposited annotated atlas (69,311 matched cells; 17,162 meningioma / 52,149 TME). (c) Tumour versus TME mean UMI on the same reference (dashed line, equality; inset lists coordinates). (d) Tumour/TME mean-UMI ratios from deposited Cell Ranger matrices versus GENCODE v26 re-quantification (dotted line, ratio = 1; Supplementary Table 4b).

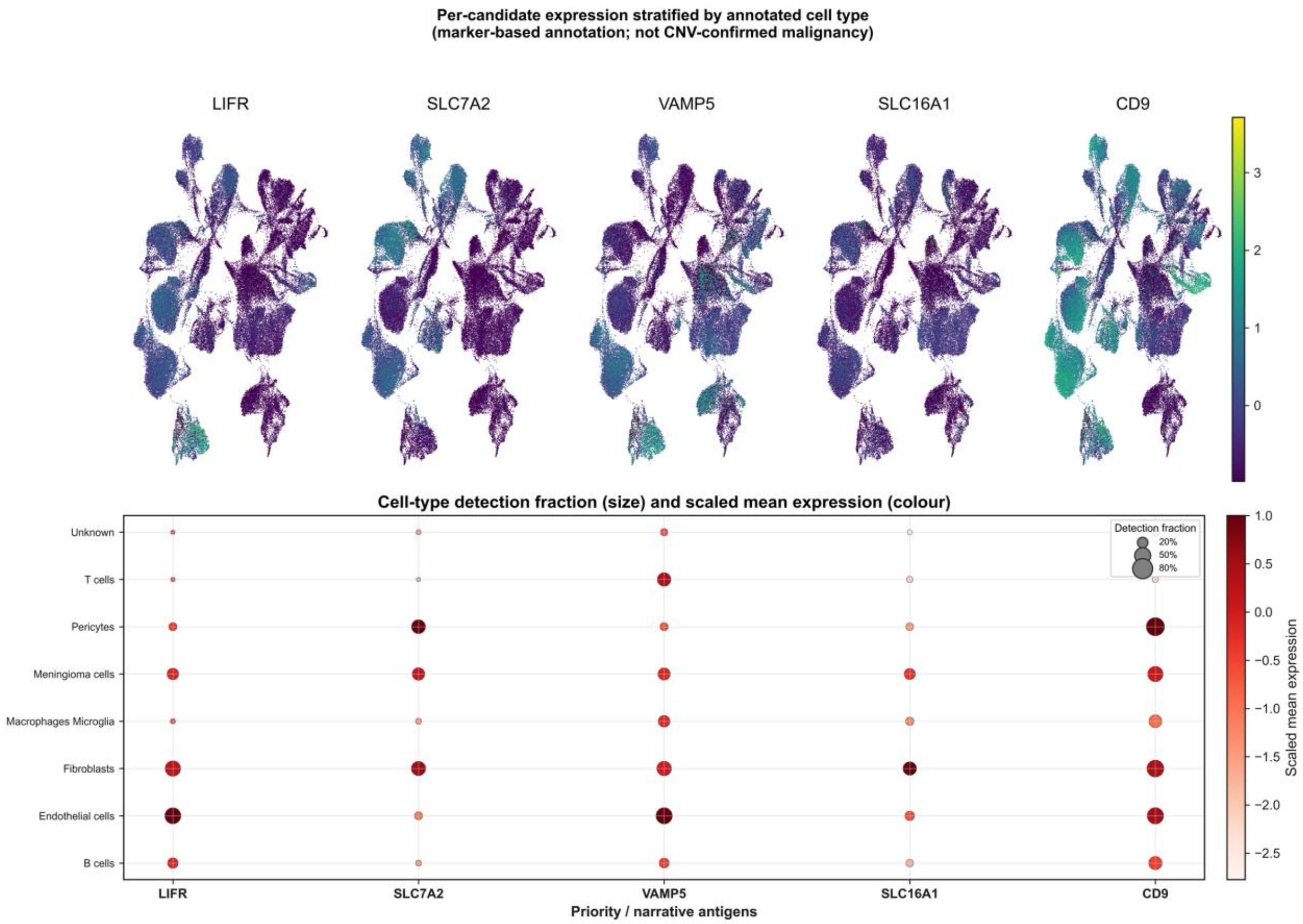

Supplementary Figure 8. Per-candidate expression of narrative antigens stratified by annotated cell type (GSE183655). Top: UMAP projections coloured by expression of LIFR, SLC7A2, VAMP5, SLC16A1 and CD9. Bottom: dot plot of detection fraction (dot size) and scaled mean expression (colour) across annotated compartments. Related numeric summaries are in Supplementary Table 7.

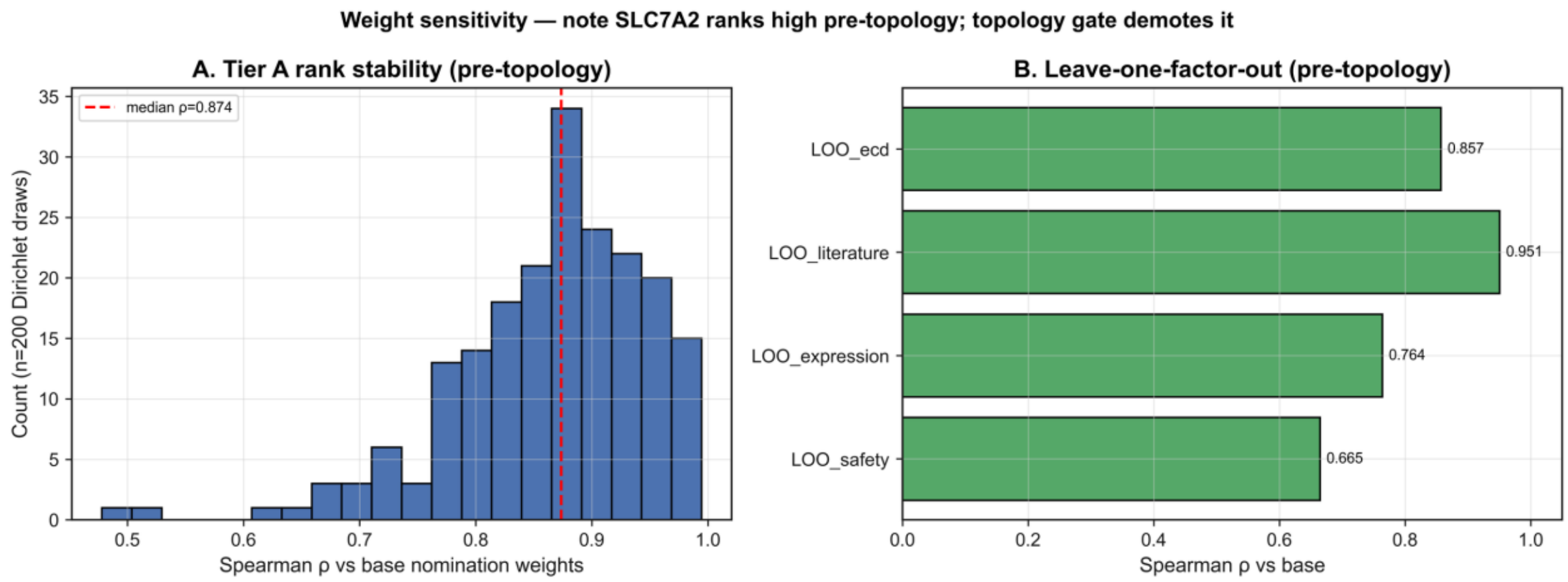

Supplementary Figure 9. Sensitivity of Tier A nomination ranking to hard-coded weights. (A) Distribution of Spearman ρ between base nomination ranking and Dirichlet weight perturbations (safety 50% / expression 25% / literature 10% / ECD 15%). (B) Leave-one-factor-out ρ versus base. Related statistics are in Supplementary Table 5.

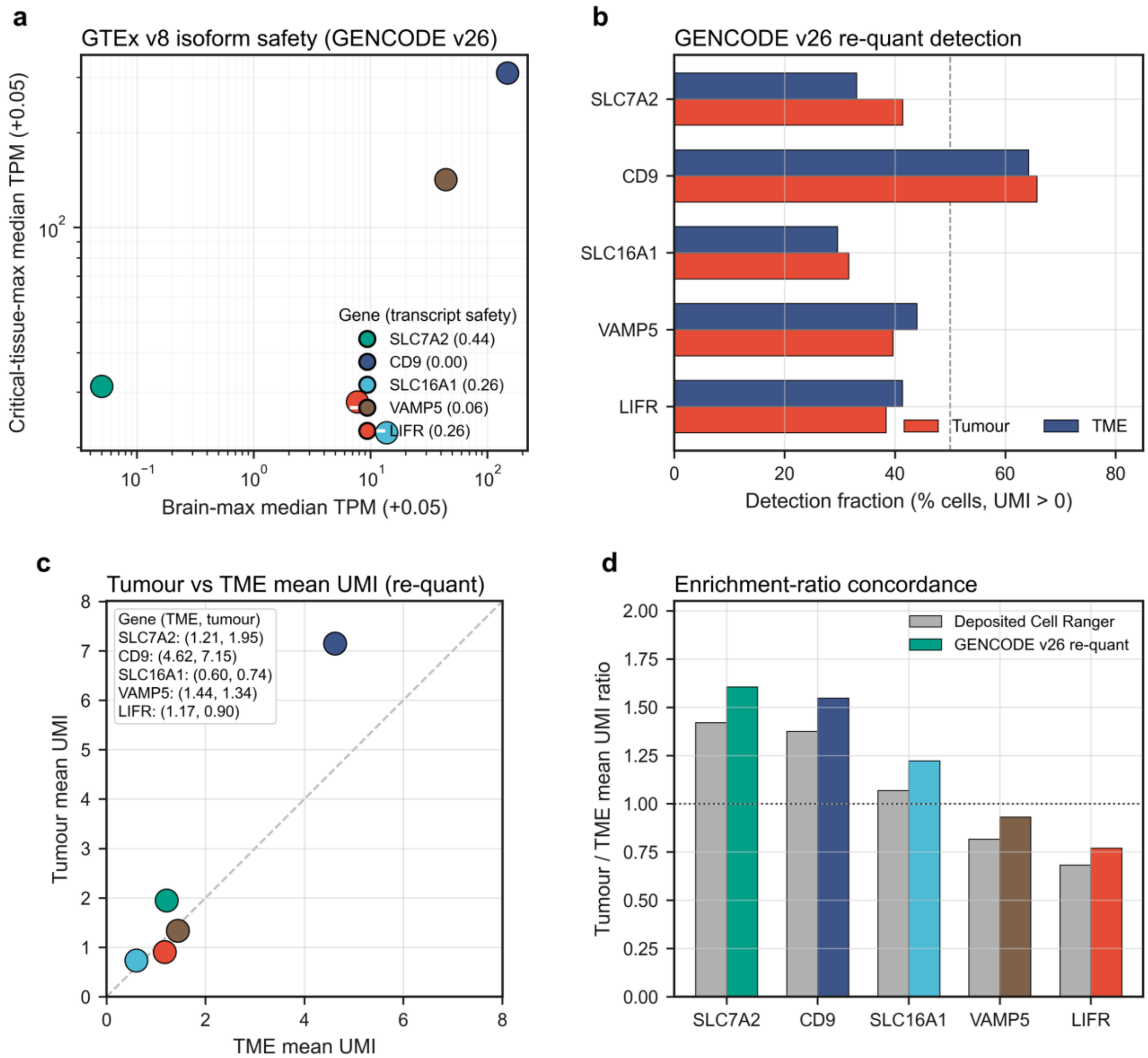

Supplementary Figure 10. GTEx v8 isoform safety and GENCODE v26 meningioma re-quantification. (a) GTEx v8 RSEM transcript TPM medians on GENCODE v26 isoforms (Supplementary Table 4a). (b–d) kallisto|bustools gene-level tumour versus TME expression after re-quantification of all 15 GSE183655 runs (Supplementary Table 4b).

1.2 Supplementary Tables

Gene	CAR-T feasibility	Safety x coverage	Gated nomination score	Expression (norm)	Literature (norm)	ECD access. (norm)	Safety (norm)	CAR-T score	Brain max TPM	Whole-body safety	Clonality (%)
LIFR	Antibody-tractable experimental priority	0.135	0.619	0.362	1.000	0.817	0.612	20.7	14.5	0.405	33.4

CD9	Filtered (ubiquitous normal-tissue protein)	0.123	—	0.600	1.000	0.862	0.349	26.5	123.4	0.231	53.2
APMAP	Filtered (ubiquitous normal-tissue protein)	0.078	—	0.252	0.300	0.735	0.382	18.1	323.9	0.253	30.6
APP	Filtered (systemic on-target risk)	0.115	—	0.380	1.000	0.902	0.340	21.2	561.1	0.225	50.9
STING1	Filtered (insufficient safety data)	—	—	0.247	0.600	0.807	0.000	18.0	—	—	32.6
VAMP5	Filtered (tail-anchored / minimal surface exposure)	0.145	—	0.344	0.300	0.776	0.601	20.3	62.2	0.398	36.5
SLC7A2	Filtered (multi-pass transporter topology)	0.245	—	1.000	0.300	0.806	0.906	36.2	4.747	0.600	40.8
C7	Filtered (ubiquitous normal-tissue protein)	0.045	—	0.027	0.700	0.818	0.337	12.7	3.648	0.223	20.3
SLC16A1	Filtered (multi-pass transporter topology)	0.137	—	0.217	0.600	0.763	0.755	17.3	20.8	0.500	27.5
BST2	Filtered (ubiquitous normal-tissue protein)	0.052	—	0.078	0.600	1.000	0.298	13.9	52.1	0.197	26.3
CD302	Filtered (low tumour coverage)	0.060	—	0.000	0.300	0.821	0.497	12.0	6.237	0.329	18.2
MCAM	Filtered (ubiquitous normal-tissue protein)	0.063	—	0.294	0.600	0.865	0.350	19.1	121.9	0.232	27.3

EVI2A	Filtered (low tumour coverage)	0.056	—	0.076	0.300	0.900	1.000	13.9	158.5	0.662	8.412

Supplementary Table 1. Complete four-dimension nomination score decomposition for all 13 Tier A antigens. Columns: gene; expression_norm (25% weight); literature_norm (10%); ecd_accessibility_norm (15%); safety_norm (50%); weighted gated score; GTEx brain TPM; GTEx peripheral TPM; GTEx whole-body component; HPA protein safety; combined safety; clonality (%); safety x coverage; therapeutic index; CNS/peripheral risk labels; car_t_feasibility; membrane_topology_class; max_ecd_loop_aa. Relative gated rank is min-max normalised within Tier A (not absolute feasibility). LIFR: gated rank 0.619, type I, loop 789 aa, safety x coverage 0.135. SLC7A2: topology-filtered; liability-laden rank 0.854; safety x coverage 0.245. Per-gene: LIFR: gated score 0.619, safety 0.41, clonality 33.4%, brain TPM 14.5 (Antibody-tractable experimental priority) SLC7A2: gated score LL-rank 0.854, safety 0.60, clonality 40.8%, brain TPM 4.7 (Filtered (multi-pass transporter topology)) VAMP5: gated score LL-rank 0.533, safety 0.40, clonality 36.5%, brain TPM 62.2 (Filtered (tail-anchored / minimal surface exposure)) SLC16A1: gated score LL-rank 0.606, safety 0.50, clonality 27.5%, brain TPM 20.8 (Filtered (multi-pass transporter topology)) CD9: gated score LL-rank 0.554, safety 0.23, clonality 53.2%, brain TPM 123.4 (Filtered (ubiquitous normal-tissue protein)) APP: gated score filtered, safety 0.23, clonality 50.9%, brain TPM 561.1 (Filtered (systemic on-target risk)) APMAP: gated score filtered, safety 0.25, clonality 30.6%, brain TPM 323.9 (Filtered (ubiquitous normal-tissue protein)) MCAM: gated score filtered, safety 0.23, clonality 27.3%, brain TPM 121.9 (Filtered (ubiquitous normal-tissue protein)) CD302: gated score filtered, safety 0.33, clonality 18.2%, brain TPM 6.2 (Filtered (low tumour coverage)) EVI2A: gated score filtered, safety 0.66, clonality 8.4%, brain TPM 158.5 (Filtered (low tumour coverage)) BST2: gated score LL-rank 0.378, safety 0.20, clonality 26.3%, brain TPM 52.1 (Filtered (ubiquitous normal-tissue protein)) C7: gated score filtered, safety 0.22, clonality 20.3%, brain TPM 3.6 (Filtered (ubiquitous normal-tissue protein)) STING1: Filtered (insufficient safety data)

Comparison	**Shared top-10**	**Total top-10**	**Overlap (%)**	**Spearman ρ**	**P-value**
GSE183655 vs GSE165341	10	10	100.0	0.934	<0.001
GSE183655 vs GSE136661	9	10	90.0	0.941	<0.001
GSE183655 vs GSE16581	9	10	90.0	0.946	<0.001
GSE165341 vs GSE136661	9	10	90.0	0.891	<0.001
GSE165341 vs GSE16581	9	10	90.0	0.873	<0.001
GSE136661 vs GSE16581	10	10	100.0	0.898	<0.001

Supplementary Table 2. Cross-dataset pairwise reproducibility statistics (six comparisons, four cohorts, n ~ 83 shared genes). Columns: comparison pair; shared top-10 count; total top-10; overlap (%); Spearman rho; P-value. Rows: GSE183655 vs GSE165341: 10/10, 100%, rho = 0.934, p < 0.001; vs GSE136661: 9/10, 90%, rho = 0.941; vs GSE16581: 9/10, 90%, rho = 0.946; GSE165341 vs GSE136661: 9/10, 90%, rho = 0.891; GSE165341 vs GSE16581: 9/10, 90%, rho = 0.873; GSE136661 vs GSE16581: 10/10, 100%, rho = 0.898. Mean overlap 93.3%; mean rho = 0.914. Inference: expression-based discovery ranking is platform-independent; CAR-T suitability requires additional surfaceome and safety layers (Table 1).

Gene	Relative MM-GBSA rank (1=best proxy)	Interface residues	Method	Note
EVI2A	1	12.0	MM-GBSA_GBn2_minimized	Ranking proxy only; absolute ΔG not reported
BST2	2	16.0	MM-GBSA_GBn2_minimized	Ranking proxy only; absolute ΔG not reported
SLC7A2	3	21.0	MM-GBSA_GBn2_minimized	Ranking proxy only; absolute ΔG not reported
CD9	4	21.0	MM-GBSA_GBn2_minimized	Ranking proxy only; absolute ΔG not reported
DCN	5	24.0	MM-GBSA_GBn2_minimized	Ranking proxy only; absolute ΔG not reported
ITGBL1	6	36.0	MM-GBSA_GBn2_minimized	Ranking proxy only; absolute ΔG not reported
APP	7	37.0	MM-GBSA_GBn2_minimized	Ranking proxy only; absolute ΔG not reported

Supplementary Table 3. MM-GBSA GBn2 relative binding rank order (Full Fab H+L; n = 7; exploratory structural proxy only). Columns: gene; relative rank (1 = most favourable delta_g_bind_per_iface_res); interface residue count; method (MM-GBSA_GBn2_minimized, CUDA). Rank order: EVI2A (rank 1), BST2 (rank 2), SLC7A2 (rank 3), CD9 (rank 4), DCN (rank 5), ITGBL1 (rank 6), APP (rank 7). Per-interface values (kcal/mol/residue, not reported as absolute DeltaG due to GBn2 force-field scaling): EVI2A -703.5 (12 interface residues); BST2 -534.0 (16 res); CD9 -404.8 (21 res); SLC7A2 -405.7 (21 res); DCN -354.6 (24 res); ITGBL1 -236.9 (36 res); APP -228.8 (37 res). Inference: MM-GBSA rank weakly correlates with gated nomination (SLC7A2 rank 4 vs nomination rank 1)—supporting exclusion of computational docking from CAR-T prioritisation. See Table 2 for HADDOCK flexref scores.

2 Supplementary Methods

2.1 Notation and general preprocessing

Scalars are denoted in italic (e.g., n cells). Vectors are bold lowercase. Matrices are bold uppercase. epsilon = 1e-10 stabilises divisions. Cohort-level normalisations for nomination scores were performed within the 83-gene validation set unless stated otherwise.

2.2 Quality control and normalisation (Modules 1-2)

Cell i is retained if: genes_i >= 200, mito_fraction_i <= 0.05, ribo_fraction_i <= 0.50.

Applied to GSE183655: 80,901 cell-called barcodes -> 80,220 retained (99.2%).

Normalised expression: x_tilde_ij = log1(1e4 * x_ij / sum_j x_ij + 1).

Highly variable genes: Scanpy highly_variable_genes (flavor=seurat_v3, n_top=2,000 for PCA; n_top=600 for beta-VAE).

2.3 Differential expression (Module 4)

Moderated t-statistic per gene g across patients: t_g = (mean_tumour,g - mean_control,g) / SE_g.

Benjamini-Hochberg FDR correction genome-wide. Significance: FDR < 0.05 and |log2FC| >= 1.0.

Consistently upregulated if significant in >= 2 of 8 patients (5,214 genes).

2.4 Tumour specificity and initial surface filter (Modules 5-6)

tumor_specificity_g = log2(mean_expr_tumour,g / mean_expr_dura,g) - max_CNS log2(GTEx_t,g).

TMHMM: >= 1 predicted transmembrane helix. GTEx pass: max_brain_TPM and specificity thresholds.

Yielded 100 tumour-specific surface proteins before curated surfaceome filter.

2.5 Multi-factor CAR-T score (Module 7)

Z-score: Z_i,f = (f_i - mu_f) / sigma_f.

combined_car_t_score = 0.20*Z_expr + 0.30*Z_spec + 0.20*Z_clon + 0.20*Z_immuno + 0.10*Z(1-escape).

clonality_i = 100 x N_expressing_i / N_total_tumour_cells.

2.6 Curated surfaceome filter (Module 25)

Tier A requires: plasma-membrane UniProt topology, ECD >= 20 aa, not ribosomal (RPS/RPL/MRPS), not nuclear/scaffold, not secreted-only without membrane anchor.

Result: 83 input -> 13 Tier A; 70 excluded.

Excluded examples: FOXC1 (nuclear, ECD=0), LDHA (cytoplasmic), MGMT (nuclear), PTGDS (secreted; GTEx brain max 2163 TPM), CPE, LGALS3, ITGBL1, DCN.

2.7 GTEx whole-body safety

GTEx Portal API v8: geneSearch with exact symbol match -> versioned GENCODE ID -> medianGeneExpression across all tissues.

brain_tier(TPM): <0.5->0.95; 0.5-5->0.80; 5-50->0.55; 50-500->0.25; >500->0.05.

gtex_whole_body = 0.30*brain_tier(max_brain) + 0.70*peripheral_tier(max_critical).

Critical tissues: Whole_Blood, Liver, Lung, Kidney_Cortex, Heart_Left_Ventricle, Spleen, Pancreas, Muscle_Skeletal, Skin_Sun_Exposed_Lower_leg, Small_Intestine_Terminal_Ileum, Colon_Transverse, Adipose_Subcutaneous.

Example SLC7A2: brain 4.7 TPM, peripheral max 89.5 TPM.

2.8 Human Protein Atlas protein cross-reference

HPA JSON: https://www.proteinatlas.org/{ensembl_id}.json (Ensembl ID without version suffix).

hpa_protein_safety integrates: IHC tissue distribution (penalty for "Detected in all"), MS blood pg/L, blood-cell RNA nTPM max, plasma-membrane confirmation, protein intensity.

CD9: "Detected in all", MS blood ~4.2M pg/L. SLC7A2: "Detected in single".

combined_safety = 0.50*gtex_whole_body + 0.50*hpa_protein_safety.

2.9 Membrane-topology tractability gate

max_ecd_loop_aa = max contiguous extracellular segment from UniProt topological domains.

Antibody-tractable: type_I_receptor (single TM + signal peptide + loop >= 100 aa), type_II_receptor (single TM + loop >= 100 aa), GPI_anchored.

Poor tractability: multi_pass_transporter (SLC/ABC families or "transporter" annotation), tail_anchored_SNARE (VAMP family), multi_pass_small_loops (>= 2 TM, max loop < 80 aa).

tm_count corrected when conflated with hpa_transmembrane_score (artifact TM=7): reassigned to TM=1 when contiguous ECD >= 200 aa with <= 2 loops.

SLC7A2: multi_pass_transporter (max loop 38 aa). LIFR: type_I_receptor (loop 789 aa). VAMP5: tail_anchored_SNARE.

2.10 CAR-T feasibility gates and nomination score

safety_x_coverage = combined_safety * (clonality_pct / 100).

Filtered from gated ranking: ubiquitous HPA; combined_safety < 0.35; clonality < 25%; poor topology.

Relative gated rank = 50% safety_norm + 25% expression_norm + 10% literature_norm + 15% ecd_norm (min-max within Tier A survivors). NOT an absolute feasibility probability.

liability_laden_rank_score: same formula for topology-filtered genes (informational only).

LIFR gated rank = 0.619; SLC7A2 liability-laden rank = 0.854 (topology-filtered). Stringent favourable: safety >= 0.55, clonality >= 50%, sxc >= 0.30.

2.11 Gradient boosting, SHAP, GCN, and beta-VAE (Modules 9, 13, 15-16)

LightGBM: objective=regression, num_leaves=31, learning_rate=0.01, feature_fraction=0.8, 1,000 rounds, early_stopping=50. XGBoost: max_depth=6, n_estimators=100, lr=0.05.

SHAP TreeExplainer: phi_j(f,x) Shapley attributions (Supplementary Fig. 2).

GCN: top 80 genes, Pearson r>0.3, hidden=64, 60 epochs, MSE loss ~240.4 (Supplementary Fig. 3).

beta-VAE: 600 HVGs, latent K=24, Adam lr=1e-3, 24 epochs, final loss ~2,986.

2.12 HADDOCK3, OpenMM, and MM-GBSA (Modules 18-24)

Epitope fragments from AlphaFold with ambiguous distance restraints (2.0 A).

HADDOCK3: rigid-body it0 (200 poses) -> caprieval -> seletop -> flexref -> caprieval.

Full Fab: molecules A (antigen), B (7JIC heavy), C (7JIC light). OpenMM: AMBER14/TIP3P, 298.15 K, 0.15 M NaCl, 2 fs timestep, ~500k steps (~1 ns); +1M steps (+2 ns) for BST2, CD9, ITGBL1.

MM-GBSA GBn2: single-point minimisation; relative rank order only (Supplementary Table 3).

HADDOCK flexref scores are exploratory and excluded from nomination ranking.

2.13 Cross-dataset statistics

overlap% = 100 x |Top10_A n Top10_B| / 10.

Spearman rho = 1 - 6*sum(d_i^2) / (n*(n^2-1)). Two-sided p via SciPy stats.spearmanr.

Six pairwise comparisons; mean overlap 93.3%; mean rho = 0.914 (Supplementary Table 2).

2.14 Literature mining and ligand-receptor screening (Modules 10-12)

PubMed/NCBI literature scores normalised within the 83-gene set (10% nomination weight). Ligand-receptor analysis (CellPhoneDB-style) screened 77 significant pairs in the tumour microenvironment; results inform immune-context interpretation but not gated nomination scores.

2.15 Software versions

Python 3.10+, Scanpy 1.9, pandas 2.x, NumPy, SciPy, LightGBM 4.x, XGBoost 2.x, SHAP 0.4x, PyTorch 2.x, OpenMM 8.x, HADDOCK3 3.x, BioPython, AlphaFold DB v6, GTEx v8, HPA v25.1, matplotlib 3.x, seaborn. Structural jobs: HADDOCK3 in WSL2; OpenMM on NVIDIA CUDA.

Reference genome and transcript-level quantification

GSE183655 analyses in this study used author-deposited Cell Ranger gene-level count matrices (Ensembl gene IDs in features.tsv) retrieved from GEO. Systemic safety used GTEx v8 gene-level median TPM (Portal API medianGeneExpression / gene TPM GCT). Preferred ENST / UniProt isoforms for LIFR, SLC7A2, CD9, VAMP5 and SLC16A1 are listed in Supplementary Table 4. We did not re-align raw FASTQs to the GTEx v8 RSEM transcript reference (GTEx_Analysis_2017-06-05_v8_RSEMv1.3.0_transcript_tpm.gct.gz) or report scRNA-seq transcript-level TPM for tumour versus microenvironment, because transcript-resolved single-cell quantifications are not provided in the analysed GEO matrices. Gene-level tumour- versus microenvironment-compartment expression for priority antigens is reported in Supplementary Figure 8 and Supplementary Table 7. FASTQ-level re-quantification against the GTEx v8 GENCODE transcript annotation is identified as future work.

Supplementary Table 4a. GTEx v8 RSEM transcript-level safety for priority antigens (GENCODE v26)

Source: GTEx_Analysis_2017-06-05_v8_RSEMv1.3.0_transcript_tpm.gct.gz with GTEx_Analysis_v8_Annotations_SampleAttributesDS.txt. Values are per-transcript median TPM within each SMTSD tissue; brain_max and critical_max are maxima over the manuscript brain and twelve critical peripheral tissue panels. Transcript safety = 0.30 × tier(brain_max) + 0.70 × tier(critical_max) with TPM tiers <1 / <5 / <20 / <50 → 1.0 / 0.7 / 0.4 / 0.2 / 0.0. ENST corrections: prior LIFR pin ENST00000263433 is PPP1R12C; prior VAMP5 pin ENST00000216268 is ZBED4; CD9 ENST00000382595 and SLC7A2 ENST00000412365 are absent from GENCODE v26. Reported IDs are GTEx-present GENCODE v26 isoforms of the intended genes.

Gene	GENCODE v26 ENST	Brain-max median TPM	Critical-max median TPM	Transcript safety
LIFR	ENST00000263409.8	7.67	27.88	0.26
SLC7A2	ENST00000398090.3	0.00	31.22	0.44
CD9	ENST00000009180.8	148.90	309.60	0.00
VAMP5	ENST00000306384.4	44.07	141.90	0.06
SLC16A1	ENST00000369626.7	13.68	22.23	0.26

Deposited Cell Ranger tumour/TME gene-level summaries for the same priority antigens (concordance with GENCODE v26 re-quant) are shown in Supplementary Figure 10d; re-quantification statistics are in Supplementary Table 4b.

Supplementary Table 4b. Priority-antigen tumour versus TME expression after GENCODE v26 kallisto|bustools re-quantification

Counts are gene-level UMIs from kallisto bus + bustools correct/count against GENCODE v26 (10x Chromium v3). Tumour = cells annotated as Meningioma cells; TME = all other annotated cells. Multi-lane SRRs sharing a GSM accession were UMI-summed per barcode before summary statistics.

Gene	GENCODE v26 ID	Tumour n	Tumour mean UMI	TME mean UMI	Tumour det.	Tumour/TME ratio
LIFR	ENSG00000113594.9	17162	0.904	1.174	0.384	0.770
SLC7A2	ENSG00000003989.17	17162	1.947	1.213	0.414	1.606
CD9	ENSG00000010278.12	17162	7.149	4.621	0.657	1.547
VAMP5	ENSG00000168899.4	17162	1.340	1.438	0.396	0.932
SLC16A1	ENSG00000155380.11	17162	0.736	0.602	0.316	1.223
